# Seeking Overlapping Neuroanatomical Alterations between Dyslexia and Attention-Deficit/Hyperactivity Disorder: A Meta-Analytic Replication Study

**DOI:** 10.3390/brainsci12101367

**Published:** 2022-10-09

**Authors:** Donato Liloia, Annachiara Crocetta, Franco Cauda, Sergio Duca, Tommaso Costa, Jordi Manuello

**Affiliations:** 1 GCS fMRI Koelliker Group, Koelliker Hospital and University of Turin, 10124 Turin, Italy; 2 FOCUS Laboratory, Department of Psychology, University of Turin, 10124 Turin, Italy; 3 Neuroscience Institute of Turin, 10043 Turin, Italy

**Keywords:** coordinate-based meta-analysis, voxel-based morphometry, ADHD, dyslexia, ALE, seed-based d mapping, replication article, reproducibility, open science, replication crisis

## Abstract

The present work is a replication article based on the paper “Are there shared neural correlates between dyslexia and ADHD? A meta-analysis of voxel-based morphometry studies” by McGrath and Stoodley (2019). In the original research, the authors used activation likelihood estimation (ALE), a technique to perform coordinate-based meta-analysis (CBMA), to investigate the existence of brain regions undergoing gray matter alteration in association with both attention-deficit/hyper-activity disorder (ADHD) and dyslexia. Here, the same voxel-based morphometry dataset was analyzed, while using the permutation-subject images version of signed differential mapping (PSI-SDM) in place of ALE. Overall, the replication converged with the original paper in showing a limited overlap between the two conditions. In particular, no significant effect was found for dyslexia, therefore precluding any form of comparison between the two disorders. The possible influences of biological sex, age, and medication status were also ruled out. Our findings are in line with literature about gray matter alteration associated with ADHD and dyslexia, often showing conflicting results. Therefore, although neuropsychological and clinical evidence suggest some convergence between ADHD and dyslexia, more future research is sorely needed to reach a consensus on the neuroimaging domain in terms of patterns of gray matter alteration.

## 1. Introduction

In their original research, McGrath and Stoodley [1] aimed to identify regions of altered gray matter shared between dyslexia and attention-deficit/hyper-activity disorder (ADHD). The conjoint investigation of these two neurodevelopmental disorders (NDDs) is not only supported by their frequently reported comorbidity, but also by shared genetic and neural pathomechanism risk factors. In this regard, converging evidence suggests that NDDs tend to report a shared etiological basis in neurodevelopment abnormality caused by complex multifactorial interactions of genetic defects, as well as of environmental, epigenetic, cognitive, and behavioral factors [2,3,4,5,6,7]. New potential metabolic targets and neuroprotective agents against NDDs, including ADHD and learning disorders, are starting to appear in the animal model research literature [7,8,9], thereby opening perspectives for future treatment.

The advent of magnetic resonance imaging (MRI) technologies has provided an unprecedented opportunity to assess the neurophysiological underpinnings of these two NDDs in vivo and noninvasively. Numerous studies about pediatrics and adults with ADHD suggest functional abnormalities in fronto-striatal and fronto-limbic systems [10,11,12,13,14] that may underlie impulsivity, hyperactivity, and inattention deficits typical of the disorder [15]. By contrast, subjects suffering from dyslexia tend to report deficits in orthographic and visuo-phonological domains, in which the occipito-temporal functional network seems to have a pivotal role [16,17,18]. From the structural point of view, abnormalities in brain morphology have been reported in both disorders, encompassing multiple areas such as the basal ganglia, cerebellum, parietal cortex, corpus callosum, prefrontal-cingulate cortex, and parieto-temporal regions [11,19,20]. However, this voluminous literature remains largely inconclusive. Moreover, only a limited pool of studies have examined neuroanatomical convergence across disorders reporting conflicting findings [21,22,23,24].

To fill this gap, McGrath and Stoodley [1] realized a coordinate-based meta-analysis (CBMA) of previously published voxel-based morphometry (VBM) findings. VBM is a widely used MRI technique in the field of human brain mapping, which allows the identification of focal differences in volume or concentration between the brains of two groups of subjects [25]. In the specific case of McGrath and Stoodley [1], the considered experiments had compared either subjects diagnosed with dyslexia against typically developing controls (TDCs), or subjects diagnosed with ADHD against TDCs. The dataset was then processed according to the CBMA approach. In general terms, this class of techniques allows us to quantify the consensus between multiple experiments based on structural or functional neuroimaging techniques [26,27]. Therefore, they represent a valuable tool for human brain mapping, offering a way to overcome the variability frequently found among single experiments [28,29]. Unlike image-based meta-analyses, which take in three-dimensional (3D) maps representing the results, CBMAs process instead the so-called “list of foci”. Here, each focus is a peak of the maximum measured cluster effect, localized through a triplet of stereotactic coordinates (x,y,z) [30]. CBMAs makes therefore possible to recover the full 3D information starting from a much sparser (but often the only available) representation of the data [31].

Among the various CBMA algorithms, McGrath and Stoodley [1] resorted to the activation likelihood estimation (ALE) technique [32,33]. Notably, this approach uses a Gaussian kernel to model the effect, adjusting the full-width half-maximum (FWHM) of the Gaussian based on the sample size of the experiment considered time to time. This means that the higher the number of subjects analyzed in the experiment, the more spatially precise (and reliable) are considered the related results, and therefore smoothed through a tighter Gaussian [34].

In the original paper, a two-step procedure was followed. First, the ALE analysis was separately applied to estimate the spatial convergence associated with each of the four possible conditions: dyslexia < TDCs; ADHD < TDCs; dyslexia > TDCs; ADHD > TDCs. The first two contrasts investigated the so-called decrease effect [35], meaning that the pathological state is associated with a reduction in gray matter; conversely, the remaining two targeted the increase effect, where an increment of gray matter is searched for instead [36]. The obtained ALE maps were thresholded using both a more conservative option (i.e., *p*_uncorrected_ < 0.001; minimum cluster size k = 50 mm^3^) and a less conservative one (i.e., *p*_uncorrected_ < 0.005; k = 50 mm^3^). In the second step, a conjunction analysis was implemented between the previously obtained dyslexia < TDCs and ADHD < TDCs maps (separately for the conservative and lenient thresholding). This allowed us to identify voxels with a statistically significant overlap between the two disorders [37]. The resulting conjunction maps were thresholded using a false discovery rate (FDR) of *p* < 0.05 (based on 5000 permutations) and k = 50 mm^3^ as above. No conjunction analysis was run for the increase condition due to lack of any overlap already at visual inspection. McGrath and Stoodley [1] did not find any overlap between dyslexia and ADHD when using ALE maps thresholded at *p*_uncorrected_ < 0.001; k = 50 mm^3^ (i.e., the most conservative option). A sole cluster in the right caudate was instead observed for *p*_uncorrected_ < 0.005; k = 50 mm^3^. In addition to the described main analyses, the authors aimed to assess the possible effect of brain volume and age on the results. Since ALE technique does not allow us to model confounding variables during the estimation of the spatial convergence, different subsets of the original dataset were extracted and separately analyzed. In the first case, only those VBM experiments that originally controlled for total brain volume or total gray matter volume were retained. In the second case, VBM experiments were divided into two groups based on the reported mean age of subjects (≤12 years; ≥18 years). Experiments were discarded if the necessary information was missing. The previously observed cluster in the right caudate was still significant in the brain volume-controlled subset. On the contrary, no overlap was found between dyslexia and ADHD in the adult subgroup. In children, a cluster of overlap was observed in the left middle frontal gyrus/supplementary motor area, for ALE map thresholded at *p*_uncorrected_ < 0.005; k = 50 mm^3^.

In the present paper, we first aimed to test the original dataset analyzed by McGrath and Stoodley [1] using a different CBMA technique. Specifically, permutation-subject images version of signed differential mapping (PSI-SDM) [38] was employed as an alternative to ALE. To the best of our knowledge, no previous study has evaluated the constancy in terms of results between the two algorithms despite substantial methodological differences. From the clinical point of view, we expected limited or completely absent neuroanatomical overlap between disorders in line with the limited available literature on the topic [1,39,40]. Given the peculiar nature of PSI-SDM, additional analyses were also performed. In fact, we directly estimated the possible interfering effect of key socio-demographic and clinical variables via voxel-wise meta-regression approach [41]. Finally, an additional analysis was made including in the dataset the nine VBM experiments with null results that were identified but excluded by McGrath and Stoodley [1].

## 2. Materials and Methods

As mentioned above, the core element of this replication attempt is the change of technique used to compute the CBMA. This implied several methodological differences that are detailed below.

### 2.1. Dataset Construction

The present replication used exactly the same set of VBM experiments analyzed by McGrath and Stoodley [1]. The lists of foci necessary as input to run any CBMA was retrieved from the Supplementary Files of the original paper. The following adjustments were necessary due to technical differences between ALE and PSI-SDM. First, while the list of foci used by ALE only contains the stereotactic coordinates (x,y,x) of the peaks of effect, the PSI-SDM method also requires a measure of effect size. Therefore, the T-value of each focus was retrieved from the original manuscripts. When missing, these were computed from Z-values or *p*-values, as implemented in the dedicated conversion utility of SDM (https://www.sdmproject.com/utilities/?show = Statistics).

To note, McGrath and Stoodley [1] designed four different main contrasts: ADHD < TDCs (23 experiments; 718 subjects; 128 foci); dyslexia < TDCs (18 experiments; 388 subjects; 81 foci of variation); ADHD > TDCs (5 experiments; 75 subjects; 21 foci); dyslexia > TDCs (5 experiments; 101 subjects; 16 foci). Because of the inclusion of T-values, PSI-SDM does not require separate inputs for gray matter increase and decrease. Therefore, only ADHD vs. TDCs (24 experiments; 1661 subjects; 149 foci) (Table 1A,B for socio-demographic and clinical details; Table S1 for methodological details), and dyslexia vs. TDCs (18 experiments; 833 subjects; 97 foci) (Table 1A,B for socio-demographic and clinical details; Table S1 for methodological details) were needed for the replication. All the input files used for the present replication are freely available as Supplementary Files.

### 2.2. Coordinate-Based Meta-Analysis via PSI-SDM

As mentioned above, this replication used the PSI-SDM method in place of the ALE originally applied by McGrath and Stoodley [1]. While ALE computes for each voxel the likelihood to find a statistically significant effect in it, based on the spatial convergence among the considered experiments [32,33], PSI-SDM evaluates the presence or absence of the effect for each brain voxel performing standard univariate voxel-wise tests [38,79]. In other words, PSI-SDM estimates the effect size. To do so, the lower and upper bounds of possible effect sizes for all voxels were evaluated with multiple imputations. Then, a map of brain alteration was reconstructed for each experiment. This was made by means of an anisotropic Gaussian kernel, which attributes higher effect sizes to the voxels that appear to be more correlated with the peak coordinates. This step is conceptually similar to the creation of the modelled activation (MA) maps in ALE, although values in the MA maps represent the likelihood of finding an effect, rather than the estimated effect size. As a further difference, in ALE the FWHM of the Gaussian kernel is changed based on the sample size of each experiment [32]. On the contrary, PSI-SDM keeps a fixed FWHM, typically set at 20 mm [38]. Continuing with the PSI-SDM procedure, the most likely effect size (based on the level of statistical significance and its standard error, the coordinates and effect sizes of the reported peaks, and the anisotropic covariance between adjacent voxels) was computed for each included experiment through the maximum likelihood techniques [80]. At this point, the obtained effect size maps of each imputation dataset were combined with a random-effects model. Then, the obtained maps were combined in a final meta-analytic map by applying Rubin’s rules. Briefly, this technique allows us to impute the overall effect sizes for each brain voxel, based on the possible different effect sizes that voxels may have had in the original unavailable 3D maps associated with each experiment. Finally, the meta-analytic map was thresholded applying a family-wise error (FWE) correction for multiple comparisons, with 1000 permutations, and the threshold-free cluster enhancement (TFCE) statistic (*p* ≤ 0.05; minimum cluster size = 10 voxels) [38].

These steps were repeated twice, for dyslexia vs. TDCs, and ADHD vs. TDCs contrasts. The PSI-SDM algorithm was set to the default parameters (i.e., VBM—gray matter modality; SDM gray matter mask; anisotropy = 1; isotropic FWHM = 20 mm; voxel size = 2 mm; number of imputations = 50).

Finally, we aimed to formally test whole-brain communalities in gray matter variation between dyslexia and ADHD by calculating the overlap between both conditions in each brain voxel. To do so, the two TFCE-corrected maps (i.e., dyslexia vs. TDCs and ADHD vs. TDCs, respectively) have to be added on top of each other and compared via the multimodal function of PSI-SDM software that calculate the most probable gray matter overlap taking into account the presence of noise in the estimation of the *p*-values of each meta-analytic map [38].

### 2.3. Impact of Socio-Demographic and Clinical Variables

While ALE does not permit the modelling of additional covariates, these can be included in PSI-SDM to perform meta-regression analyses [41]. First, in order to test the hypothesis originally made by McGrath and Stoodley (i.e., the influence of subjects’ age for ADHD and dyslexia on gray matter differences), one variable was created to account for age, taking the mean age of the clinical groups as reported in Table 1 of McGrath and Stoodley [1], as to obtain the overall mean age for each experiment. VBM experiments that did not report these data were excluded from this specific analysis. To note, the impact of age was separately tested for ADHD and dyslexia datasets. Therefore, the age variable was treated as independent variable in a univariate linear regression over the voxel-wise magnitude of gray matter brain alteration. The potential impact of biological sex (percentage of male), full-scale intelligence quotient (FSIQ; mean score), and medication (percentage of medicated subjects at the scan session) was also explored for ADHD and dyslexia datasets when at least 50% of the experiments for each dataset provided the required information.

The results of the meta-regressions were thresholded at *p*_uncorrected_ < 0.0005 and minimum cluster size = 10 voxels, as suggested by the SDM team to reach the optimal balance between specificity and sensitivity [41].

### 2.4. Brain Volume Sub-Analysis

McGrath and Stoodley [1] also tested the possible confounding effect of total brain volume, or gray matter volume. To do so, they reduced the dataset to the group of experiments that explicitly corrected results to account for the volumetric difference between the clinical and control groups. Since this kind of hypothesis can’t be tested by means of a meta-regression, we followed the same original approach, but using PSI-SDM in place of ALE to analyze the identified subset.

### 2.5. Additional Analysis: Impact of Null Experiments

Knowing that some attempts to find a given effect of interest have yielded null results is of great relevance when running a CBMA [81,82]. Quantifying the exact number of null experiments is generally hard, as formalized in the so-called “file-drawer effect” bias [31,81,82]. Nonetheless, McGrath and Stoodley [1] identified nine of them during their literature search. However, it is not possible to process null experiments with the ALE method, as this would result into empty MA maps that can’t be modelled by the algorithm. On the contrary, PSI-SDM allows the consideration of null results as well. Therefore, an additional analysis was performed after the inclusion of those nine experiments into the dataset, correctly divided between dyslexia and ADHD (see also Table 2 for demographic and clinical details; Table S2 for methodological details).

## 3. Results

We aimed to replicate each of the analyses described in McGrath and Stoodley [1]. Moreover, we performed some additional analyses that the authors of the original work had been unable to carry out due to methodological limitations.

### 3.1. Gray Matter Variations in ADHD Groups

When looking at the gray matter decrease effect associated with ADHD (i.e., ADHD < TDCs) McGrath and Stoodley [1] found 11 clusters, encompassing the left frontal gyrus, the right superior orbitofrontal gyrus, the right medial frontal gyrus, the right gyrus rectus, the bilateral cingulate gyrus, the left precentral gyrus, the left superior temporal gyrus, the right putamen, the left amygdala, and the right caudate head. The increase effect (i.e., ADHD > TDCs) was observed instead in 18 clusters, covering the left superior frontal gyrus, the right precentral gyrus, the bilateral postcentral gyrus, the right supplementary motor area, the left paracentral lobule, the left posterior cingulate gyrus, the bilateral precuneus, the left cuneus, the right mid-occipital gyrus, the left medial dorsal nucleus of the thalamus, and the left insula. As highlighted by McGrath and Stoodley, these results were obtained applying a threshold of *p*_uncorrected_ < 0.001. For the sake of clarity, it should be mentioned that the use of the uncorrected thresholding is no longer recommended in the ALE field [33]. Therefore, any interpretation of the results obtained for individual disorders should be made with caution. The conjunction analysis was FDR corrected instead, in line with current guidelines.

Since PSI-SDM, as mentioned in the Methods section, can analyze decrease and increase effects together, the replication of this step consisted of a unique ADHD vs. TDCs contrast. Our results showed no effect applying a TFCE *p* ≤ 0.05; minimum cluster size = 10 voxels thresholding. Five clusters of decrease effect were instead observed at the intermediate step of the analyses when the *p*_uncorrected_ < 0.005; minimum cluster size = 10 voxels threshold was used. Although it is not infrequent in literature to describe results surviving this lenient thresholding, the current recommended statistical standard is TFCE [38]. Therefore, we have decided to include those less robust results in the Supplementary Materials only (Table S3 and Figure S1, respectively), for the sake of clarity and completeness.

### 3.2. Gray Matter Variations in Dyslexia Groups

When looking at the gray matter decrease effect associated with dyslexia (i.e., dyslexia < TDCs) McGrath and Stoodley [1] found 12 clusters, localized over the right superior frontal gyrus, the right orbitofrontal gyrus, the bilateral supramarginal gyrus, the bilateral superior temporal gyrus, the left middle temporal gyrus, the right inferior occipital gyrus, the bilateral caudate body, the left medial dorsal nucleus of the thalamus, the left insula, and the left lobule VI in the cerebellum. The increase effect (i.e., dyslexia > TDCs) was observed instead in 13 clusters, encompassing the bilateral medial superior frontal gyrus, the right precentral gyrus, the right supplementary motor area, the right paracentral lobule, the right precuneus, the left inferior parietal lobule, the bilateral superior temporal gyrus, the left middle temporal gyrus, and the left crus I in the cerebellum. As for ADHD, a threshold of *p*_uncorrected_ < 0.001 was used.

In our replication, no effect was found for the contrast dyslexia vs. TDCs, neither at TFCE *p* ≤ 0.05 nor at *p*_uncorrected_ < 0.005 (Table S4).

### 3.3. Common Gray Matter Variations in Dyslexia and ADHD Groups

Although McGrath and Stoodley [1] found wide patterns of effect for both ADHD and dyslexia, the conjunction analysis highlighted no convergence between the two neurodevelopmental conditions when considering decrease ALE maps thresholded at *p*_uncorrected_ < 0.001. When the authors lowered the threshold to *p*_uncorrected_ < 0.005 a sole cluster of decrease in the right caudate survived FDR *p* < 0.05 (k = 50 mm^3^; 5000 permutations) correction. No conjunction analysis for the increase effect was carried out instead.

Concerning our results, since no effect was found in the main PSI-SDM about dyslexia, it was not possible to compute the conjunction analysis, neither at TFCE *p* ≤ 0.05 nor at *p*_uncorrected_ < 0.005 thresholding.

### 3.4. Additional Results: Impact of Null Experiments

As described in the Methods section, PSI-SDM allows us to also model experiments that found null results. Therefore, we repeated the analyses described above after having complemented the database with the null experiments reported in McGrath and Stoodley (2019). This was an additional analysis, not implemented in the original research due to methodological constrain. Concerning ADHD, still no effect was found at TFCE *p* ≤ 0.05, in line with what observed for the original database. Coherently, four clusters of decrease effect were observed at *p*_uncorrected_ < 0.005 threshold (Table S5 and Figure S2). The inclusion of the null experiments did not affect dyslexia that still showed no cluster of effect at any level of thresholding (Table S6). As in the case of the original database, it was not possible to complete the conjunction analysis due to the lack of effect at previous stages.

### 3.5. Impact of Socio-Demographic and Clinical Variables

In order to evaluate the potential effect of age, McGrath and Stoodley [1] created and separately analyzed subsets of experiments depending on the mean age of the sample. When focusing on the decrease effect in adult groups (i.e., mean age > 18 years), the conjunction analysis showed no convergence between ADHD and dyslexia, irrespective of the threshold level applied to the ALE maps. The same happened for children groups based on the ALE maps thresholded at *p*_uncorrected_ < 0.001. When using the more lenient *p*_uncorrected_ < 0.005, a cluster of convergent decrease was observed in the left middle frontal gyrus and supplementary motor area. The authors did not consider the increase effect for this analysis due to the paucity of data. As explained in the Methods section, we decided to leverage on the features of PSI-SDM and perform a meta-regression, rather than separately analyzing the subset. This was in fact the most direct way to test the potential effect of age, as originally hypothesized by McGrath and Stoodley [1]. Our results showed that no effect of age was found at *p*_uncorrected_ ≤ 0.0005 either in ADHD or dyslexia.

Additionally, meta-regression analyses about biological sex and medication indicated no significant effect in both ADHD and dyslexia VBM findings. FSIQ meta-regression was not performed instead due to a large amount of unavailable data about the pertaining variable (Table 1).

### 3.6. Brain Volume Sub-Analysis

In order to evaluate the possible effect of total brain volume, McGrath and Stoodley [1] reduced the analysis to the subset of experiments that explicitly corrected the results for the volumetric difference between the clinical and control group. Even in this condition, convergence was observed in the sole cluster in the right caudate, based on the less conservative version of the maps (i.e., *p*_uncorrected_ < 0.005).

**Figure 1:**
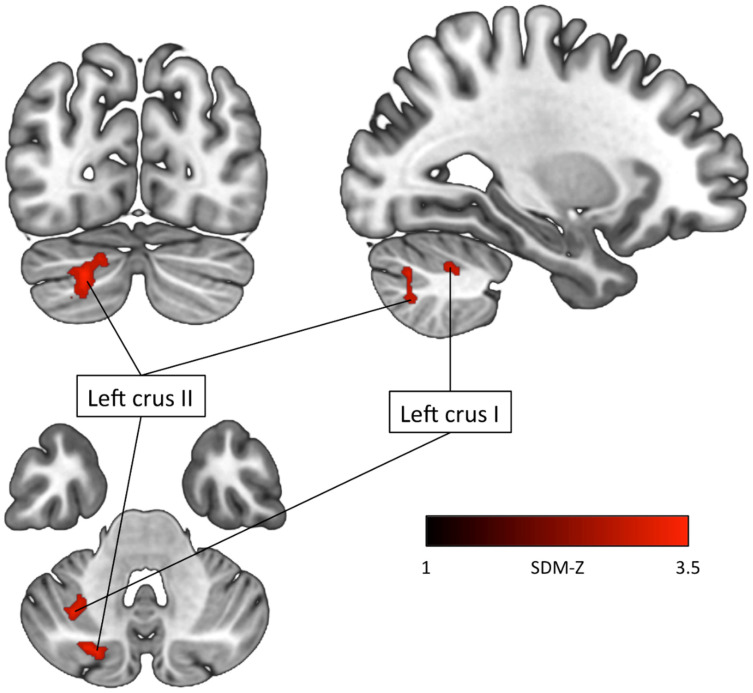
Brain cluster of gray matter reduction in subjects with attention-deficit/hyper-activity disorder compared to typically developing controls (brain volume sub-analysis). Results are TFCE-based FWER corrected at 0.05. The PSI-SDM findings are visualized as coronal, sagittal, and axial slices (2-D cortical, subcortical, and cerebellar view).

In our replication, as in the case of using the whole dataset, it was not possible to perform the conjunction analysis at TFCE *p* ≤ 0.05 since no significant effect was found for dyslexia at that threshold. The only two clusters that survived at this corrected thresholding, based on the subset for ADHD, were localized in the left crus I and crus II of the cerebellum (Table 3 and Figure 1). It is important to note that no significant heterogeneity of effect size (i.e., I^2^ = 4.5% for the peak 1; I^2^ = 17.9% for the peak 2) and no obvious publication bias (i.e., Egger’s test *p* = 0.6 for the peak 1; *p* = 0.6 for the peak 2) [80] were found for these brain volume related findings.

At the uncorrected level of statistical significance (*p* < 0.005), we found three clusters of gray matter decrease in dyslexia (Table S7; Figure S3) and 13 clusters of gray matter decrease in ADHD (Table S8; Figure S4) respectively, when accounting for brain volume. For the sake of completeness, we ran the conjunction analysis comparing the two maps at the uncorrected level. Results showed no common brain area of variation.

## 4. Discussion

In the present study, we aimed to replicate the original VBM meta-analysis by McGrath and Stoodley [1], using PSI-SDM in place of ALE as a method to carry out the analyses. Overall, the current attempt confirmed a limited overlap between the alteration correlates of ADHD and dyslexia. This was primarily due to the lack of significant effects for dyslexia that prevented the execution of the conjunction analysis. Even for ADHD, the only main results were obtained at uncorrected thresholding, and should therefore be interpreted with caution.

Nevertheless, this outcome was not completely surprising. As correctly stated by McGrath and Stoodley [1] throughout their work, the magnitude of the identified effect was limited. In fact, the conjunction analysis highlighted the only cluster in the right caudate, and only when comparing maps with the more lenient and very liberal thresholding (i.e., *p*_uncorrected_ < 0.005). The authors did not test their results with more conservative correction thresholds, such as the false discovery rate [92], voxel- or cluster-level FWE [33]. Therefore, any consideration about the behavior of the data in that scenario would be speculative [93]. As a further and related aspect, it should be noted that the number of experiments originally included in the various analyses was very close to the lower bound recommended in the ALE literature [33,94,95]. In similar cases, the stability of the results can be limited, and findings can be driven by single experiments [31,93,94]. In light of these considerations, the opposite outcomes we found could be more related with the size of the dataset than with the influence of methodological differences between ALE and PSI-SDM.

Although for some of the additional analyses we performed the null experiments for were included, the particular nature of these studies did not really contribute to expand the dataset. On the contrary, the effect of considering null results is rather to further increase the threshold to be reached by the remaining experiments. In line with this, one cluster of gray matter decrease in ADHD was lost after the inclusion of the seven null experiments. In our analyses, the only two clusters surviving the TFCE corrected *p* ≤ 0.05 thresholding were found in the left cerebellar crus II and crus I, based on the subset of ADHD experiments that accounted for total brain volume differences. Although the involvement of the cerebellum in this disorder was not reported by McGrath and Stoodley [1], this is well described in ADHD literature [39,96,97,98,99]. The fact that, in our replication, the alteration of the cerebellum only emerged in the sub-analysis could be due to the homogenization induced through the selection process. In fact, an effect of excluding the experiments that had not taken into account differences in total brain volume could be to retain more similar brains, in spatial terms. This could in turn increase the chance of finding convergence among the various experiments, therefore surviving to statistical thresholding. On the other hand, it should also be considered that when reducing the number of experiments analyzed, the chance to find some significant results increases, in virtue of reduced variance [93].

A very strict interpretation of the paucity of significant results in our replication would be that neither ADHD nor dyslexia are consistently associated with a pattern of gray matter alteration in the brain. This stance is coherent with a recent ALE cluster-level FWE corrected study by Samea et al. [40] on pediatric subjects with ADHD. By contrast, prior CBMAs described significant, albeit largely different, patterns of neuroanatomical alteration in dyslexia [100,101,102]. The discrepancy in VBM findings between current and early meta-analyses could be explained by a number of factors. First, the CBMAs of Linkersdörfer et al. [100] and Richlan et al. [101] analyzed small datasets due to the limited availability of appropriate data (i.e., nine experiments for a total of 62 gray matter decrease foci and nine experiments for a total of 45 gray matter decrease/increase foci, respectively), hence prone to type I error [93]. Second, Yan et al. [102] evaluated the neuroanatomical landscape of dyslexia from a cross-linguistic writing perspective, partitioning the current VBM literature about disorder in two datasets, namely the alphabetic language (21 experiments) and morpho-syllabic (6 experiments) groups. Third, Richlan et al. [101] and Yan et al. [102] used the effect-size version of SDM at uncorrected level; Linkersdörfer et al. [100] used the ALE instead. While these CBMA methods test the spatial convergence across coordinates, our PSI-SDM approach conducts standard univariate voxel-wise tests [38,79]. From a methodological point of view, this means that we were able to overcome certain spatial drawbacks which may have decreased the statistical power of the meta-analysis, leading to either spuriously conservative or spuriously liberal results [38,79,103]. As a further relevant note, the current lack of consensus would be further reinforced by the complex and not fully understood nature of these neurodevelopmental multi-faceted disorders. For example, some authors have suggested that both ADHD and dyslexia might not be discrete entities but, rather, their symptomatology occurs on a continuum [104,105,106,107,108]. Moreover, medical comorbidity in these clinically heterogeneous conditions is frequent [109,110]. In this regard, we note that 15 out of 31 original VBM experiments about ADHD (i.e., the 48% of the dataset) have recruited at least one subject with other psychiatric and neurological disorders (Table 1) [1]. This aspect adds inevitable heterogeneity to the meta-analytic sample.

A further aspect to be mentioned is the role of the gray matter increase. While some clusters of decrease were found at the uncorrected level of thresholding, no increase was detected in our replication. On the contrary, McGrath and Stoodley [1] found several clusters of increase in both ADHD and dyslexia. As discussed in Mancuso et al. [36], the biological meaning of the increment of gray matter in the pathological brain remains elusive, as well as its relationship with the opposed phenomenon of decrease. However, the divergent findings could be explained by the different approach followed by ALE and PSI-SDM. While the former analyses increase and decrease separately, PSI-SDM processes the two effects together. In virtue of this, if the prevalence of experiments reports the decrease of a given brain region, this could hide the presence of some increase effect in that same region. The two directions could also be counterbalancing, showing zero effect in total. Since it is known that increase effect is less represented in literature than decrease one [35,36], the absence of significant increase results should always be considered with caution.

### Limitations and Future Directions

Disorder-specific issues and clinical heterogeneity aside, we should note that the CBMA approach in general, and PSI-SDM technique in particular, have some limitations. By definition, coordinate-based techniques have a limited accuracy because they only consider significant foci (i.e., x,y,z peak values) instead of the entire voxel-wise statistic parametric maps [30]. However, we observe that this procedure is standardized in the field and capable of reducing the probability of making spatial errors [32,41]. Second, although McGrath and Stoodley [1] identified nine VBM studies with null result experiments about ADHD and dyslexia, we cannot exclude that this research topic is affected by the publication bias against null or contra-evidence results (i.e., file-drawer problem) [30,31]. Third, exploratory meta-regression analyses did not find a significant impact of some key socio-demographic and clinical variables on published findings in both clinical conditions of interest. It is necessary to note that these results are based on a limited number of eligible experiments and, therefore, should be taken with caution and deserves future attention. Fourth, in performing the SDM-PSI analyses we cannot rule out that taking into account a few experiments may slightly bias effect sizes towards zero, even though simulations made by the SDM team with the maximum likelihood/multiple imputation algorithm have already shown that this kind of bias is almost negligible [38]. Lastly, although the meta-analytic approach has permitted a quantitative synthesis of over 20 years of research about the topic, the cross-sectional nature of the data hampers the possibility to characterize possible disorder-specific and common patterns of neuroanatomical variation from a developmental perspective. In this regard, future longitudinal studies scanning the same individuals across the lifespan, along with new reproducible data analytic pipelines, may open new lines of research able to propose new neuroimaging-based targeted interventions.

## 5. Conclusions

Here, we aimed to replicate the important findings pertaining the existence of brain regions undergoing gray matter alteration in association with both ADHD and dyslexia reported in the McGrath and Stoodley study [1]. Using a different state-of-the-art meta-analytic method and additional statistical procedures, we found no significant alteration overlap between these two neurodevelopmental conditions. These results remained unchanged under the addition of nine experiments not included in the original analyses. Furthermore, we have argued that the evidence for the existence of socio-demographic and clinical confounding effects on published findings is not convincingly demonstrated. Despite common genetic, environmental, cognitive, and pathomechanism risk factors between these two NDDs, current outcomes support the existence of a marked distinction at the neural level, which may be useful for a clinical point of view especially when comorbidity is present. In sum, we believe that the overall replication of the original study may be a further step forward that will help us to find precise neural markers of these neurodevelopmental conditions.

## Figures and Tables

**Table 1:** Voxel-based morphometry experiments included in the original coordinate-based meta-analysis by McGrath and Stoodley [1]: demographic and clinical details for the attention-deficit/hyper-activity disorder (**A**) and dyslexia (**B**) datasets.

VBM Experiments Included in the Original Coordinate-Based Meta-Analysis	Clinical Group							Control Group					Brain Volume Analysis	Comorbidity with Dyslexia or ADHD Noted in Exclusion Criteria	Co-Morbid Disorders Reported in Sample
	N	% Male	Mean Age (yrs)	Age SD (yrs)	Age Range (yrs)	FSIQ	Medication	N	% Male	Mean Age (yrs)	Age SD (yrs)	Age Range (yrs)			
**(A) ADHD**															
Ahrendts et al., 2011 [42]	31	65%	31.2	9.7	18–55	N/A	0%	31	65%	31.5	8.6	19–52	yes	LD, psychiatric disorder, abuse/dependency	Anxiety
Bonath et al., 2018 [43]	18	100%	13.6	1.7	11–17	N/A	55.6%	18	100%	14.1	1.3	11–17	yes	-	1 ODD
Bralten et al., 2016 [44]	307	68%	17.1	3.4	8–26	97.08	88.6%	196	51%	16.7	3.1	9–24	no	LD, psychiatric disorder, abuse/dependency	-
Brieber et al., 2007 [45]	15	100%	13.1	1.4	10–16	N/A	66.7%	15	100%	13.3	1.8	10–16	yes	-	-
Carmona et al., 2005 [46]	25	84%	10.8	3.0	N/A	>80	100%	25	84%	11.2	3.2	N/A	yes	-	11 anxiety, 2 MDD, 4 phobias, 6 tics, 7 obsessions
He et al., 2015 [47]	37	100%	9.9	2.4	7–16	>90	0%	35	100%	10.7	2.6	8–15	yes	-	-
Iannaccone et al., 2015 [48]	20	61%	14.5	1.5	12–16	108.46	65%	20	50%	14.8	1.2	12–16	yes	-	2 affective disorder, 3 AD, 3 anxiety/phobia, 2 dyscalculia, 2 CD
Johnston et al., 2014 [49]	34	100%	12.5	2.3	N/A	N/A	29.4%	34	100%	13.2	1.0	N/A	no	-	1 dyslexia, 3 ODD/CD
Kappel et al., 2015 (adults) [50]	16	94%	23.5	4.1	19–31	N/A	0%	20	100%	23.7	3.4	N/A	no	-	2 alcohol abuse, 1 multiple drug abuse
Kappel et al., 2015 (children) [50]	14	71%	9.8	1.3	8–12	N/A	0%	10	80%	11.0	1.3	N/A	no	-	-
Kaya et al., 2018 [51]	19	71%	10.3	2.0	7–14	N/A	0%	18	67%	10.2	2.0	6–14	no	-	-
Kobel et al., 2010 [52]	14	100%	10.4	1.3	9–13	N/A	100%	12	100%	10.9	1.6	9–13	yes	-	3 OCD-CD, 2 GAD, 2 OCD-GAD
Kumar et al., 2017 [53]	18	100%	9.6	1.8	7.5–13	N/A	0%	18	100%	9.7	1.9	7.5–13	yes	LD, psychiatric disorder, abuse/dependency	-
Lim et al. 2013 [54]	29	100%	13.8	1.8	10.5–16.5	N/A	20%	29	100%	14.4	2.5	10.7–17.9	no	LD	-
McAlonan et al., 2007 [55]	28	100%	9.9	2.0	6–13	N/A	100%	31	100%	9.6	1.8	6–13	yes	-	16 OCD, 2 CD
Montes et al., 2010 [56]	20	50%	29.0	4.0	25–35	N/A	N/A	20	50%	27.6	2.6	25–35	no	-	-
Moreno-Alcazar et al., 2016 [57]	44	66%	31.6	11.4	18–65	N/A	65.9%	44	66%	32.6	10.6	18–65	no	-	-
Overmeyer et al., 2001 [58]	18	83%	10.4	1.7	8–13	N/A	N/A	16	94%	10.3	2.2	7–14	yes	LD, psychiatric disorder, abuse/dependency	1 dyslexia, 2 ODD, 2 CD
Roman-Urrestarazu et al., 2016 [59]	49	76%	22.2	0.7	20–24	96.4	0%	34	50%	22.9	0.4	20–24	no	-	-
Sasayama et al., 2010 [60]	18	72%	10.6	2.9	6–16	90.05	0%	17	71%	10.0	2.4	6–14	yes	LD, psychiatric disorder, abuse/dependency	6 ODD, 4 CD
van Wingen et al., 2013 [61]	14	100%	32.0	7.0	N/A	N/A	0%	15	100%	37.0	6.0	N/A	yes	-	-
Villemonteix et al., 2015 (med naïve group) [62]	33	55%	10.3	1.4	7.3–12.9	N/A	0%	24	50%	10.0	1.2	7.3–12.9	no	-	-
Villemonteix et al., 2015 (med group) [62]	20	80%	10.4	1.4	7.3–12.9	N/A	100%	24	50%	10.0	1.2	7.3–12.9	no	-	-
Yang et al., 2008 [63]	57	61%	11.1	N/A	N/A	97.9	87.7%	57	60%	11.7	N/A	N/A	yes	-	5 LD, 14 ODD, 1 tic, 1 GAD
*Totals, sample size, averages*	898	76%	16.5	-	-	-	-	763	71%	16,6	-	-	-	-	-
**(B) Dyslexia**															
Brambati et al., 2004 [64]	10	50%	31.6	N/A	13–57	107,1	N/A	11	45%	27.4	N/A	14–55	yes	Psychiatric disorder	-
Brown et al., 2001 [65]	16	100%	24.0	5.0	18–40	>90	N/A	14	100%	N/A	N/A	N/A	no	ADHD	-
Eckert et al., 2005 [66]	13	100%	11.4	0.7	10.1–12.7	N/A	N/A	13	100%	11.3	0.7	10.1–12.7	yes	Psychiatric disorder	-
Evans et al., 2013 (male adults) [67]	14	100%	42.9	10.4	N/A	108.0	0%	14	100%	41.1	9.0	N/A	yes	Psychiatric disorder	-
Evans et al., 2013 (female adults) [67]	13	0%	34.0	11.6	N/A	99.6	0%	13	0%	27.9	9.7	N/A	yes	Psychiatric disorder	-
Evans et al., 2013 (male children) [67]	15	100%	9.6	1.3	N/A	101.7	0%	15	100%	8.3	2.1	N/A	yes	Psychiatric disorder	-
Evans et al., 2013 (female children) [67]	17	0%	10.1	2.1	N/A	101.9	0%	17	0%	9.1	3.0	N/A	yes	Psychiatric disorder	-
Hoeft et al., 2007 [68]	19	53%	14.4	1.9	7–16	N/A	N/A	19	53%	14.4	2.4	7–16	yes	Psychiatric disorder	-
Jednorog et al., 2015 [69]	130	57%	10.3	0.9	8.5–13.7	>85	N/A	106	48%	10.2	0.9	8.5–13.7	yes	ADHD	-
Kronbichler et al., 2008 [70]	13	100%	15.9	0.8	14–16	N/A	N/A	15	100%	15.5	0.6	14–16	yes	Psychiatric disorder	-
Liu et al., 2013 [71]	18	72%	11.8	0.6	10.4–12.6	>90	0%	18	83%	11.8	0.3	11.3–12.6	yes	ADHD	-
Silani et al., 2005 [72]	32	100%	24.4	5.0	N/A	110	N/A	32	100%	26.3	5.0	N/A	no	-	-
Siok et al., 2008 [73]	16	50%	11.0	0.5	10.2–11.6	N/A	N/A	16	81%	11.0	0.6	9.11–12.4	yes	ADHD	-
Steinbrink et al., 2008 [74]	8	75%	20.1	3.9	N/A	N/A	N/A	8	75%	23.7	4.3	N/A	yes	Psychiatric disorder	-
Tamboer et al., 2015 [75]	37	16%	20.6	1.5	N/A	N/A	N/A	57	12%	20.3	1.1	N/A	yes	ADHD	-
Vinckenbosch et al., 2005 [76]	13	100%	N/A	N/A	17–30	N/A	N/A	10	100%	N/A	N/A	17–30	yes	ADHD	-
Xia et al., 2016 [77]	24	58%	12.5	0.7	10–15	>80	N/A	24	50%	12.5	0.4	10–15	no	Psychiatric disorder	-
Yang et al., 2016 [78]	9	33%	12.6	0.6	N/A	N/A	N/A	14	43%	12.3	1.0	N/A	yes	ADHD	-
*Totals, sample size, averages*	417	61%	16.4	-	-	-	-	416	57%	16.5	-	-	-	-	-

ADHD, attention-deficit/hyper-activity disorder; anxiety, anxiety disorders; CD, conduct disorder; FSIQ, full-scale intelligent quotient; GAD, generalized anxiety disorder; LD, learning disability; MDD, major depressive disorder; N, sample size; N/A, data not available; ODD, oppositional defiant disorder; psychiatric, no history of psychiatric disorders; SD, standard deviation; yrs, years; VBM, voxel-based morphometry.

**Table 2:** Voxel-based morphometry experiments with null results and therefore not included in the original coordinate-based meta-analysis by McGrath and Stoodley [1]: demographic and clinical details for the attention-deficit/hyper-activity disorder (**A**) and dyslexia (**B**) datasets.

VBM Experiments Not Included in the Original Coordinate-Based Meta-Analysis	Clinical Group							Control Group					Comorbidity with Dyslexia or ADHD Noted in Exclusion Criteria	Co-Morbid Disorders Reported in Sample
	N	% Male	Mean Age (yrs)	Age SD (yrs)	Age Range (yrs)	FSIQ	Medication	N	% Male	Mean Age (yrs)	Age SD (yrs)	Age Range (yrs)		
**(A) ADHD**														
Amico et al., (2011) [83]	20	75%	33.6	10.2	N/A	N/A	N/A	20	75%	34.7	10.7	N/A	PD	6 MDD, 7 depressive episodes
Depue et al., (2010) [84]	31	61.10%	20	1.7	N/A	114.2	77.4%	21	38.90%	19.3	1.1	N/A	LD or psychiatric disorder	-
Maier et al., (2015) [85]	131	48.90%	34.5	10.0	18–58	113.1	0%	95	47.40%	37.7	10.5	18–58	Neurological disorder	History of depression and/or psychopharmacotherapy
Onnink et al., (2013) [86]	119	38.70%	36.29	10.90	N/A	107.5	69%	107	42.10%	36.9	11.54	N/A	Neurological disorder or psychiatric condition	-
Saad et al., (2017) [87]	34	73.50%	13.28	2.75	8–17	N/A	0%	28	68%	13.09	2.63	8–17	-	ODD
Seidman et al., (2011) [88]	24	51%	37,3	12.6	18–59	116.0	87.5%	54	46%	34.3	11.3	18–59	Neurological disorderabuse or dependence	LD, MDD
Villemonteix et al., (2015) [89]	33	54.60%	10,1	1.3	7.9–12.9	105.6	0%	27	48.10%	10.1	1.3	7.9–12.9	LD, psychiatric disorder or neurological disorder	-
*Totals, sample size, averages*	392	51%	28.4	-	-	-	-	352	44%	31.6	-	-	-	-
**(B) Dyslexia**														
Eckert et al., (2016) [90]	164	60%	10.8	2.59	N/A	N/A	N/A	129	60%	10.8	2.73	N/A	LD of a logographic written language system	-
Pernet et al., (2009) [91]	38	89.50%	27.3	7.9	N/A	N/A	0%	39	89.70%	27.8	5.8	N/A	Neurological, psychiatric disorder or sensory deficits	-
*Totals, sample size, averages*	202	66%	13.9	-	-	-	-	168	67%	14.7	-	-	-	-

ADHD, attention-deficit/hyper-activity disorder; FSIQ, full-scale intelligent quotient; LD, learning disability; MDD, major depressive disorder; N, sample size; N/A, data not available; neurological disorder, no history of neurological disorders; ODD, oppositional defiant disorder; psychiatric, no history of psychiatric disorders; SD, standard deviation; yrs, years; VBM, voxel-based morphometry.

**Table 3:** Brain clusters of gray matter variation in attention-deficit/hyper-activity disorder compared with typically developmental controls at TFCE corrected *p* ≤ 0.05 and minimum cluster size = 10 voxels (brain volume sub-analysis).

Region	MNI Coordinate			SDM	*p* ≤ 0.05	Voxels	Cluster Breakdown
	x	y	z	*Z* Score	(Corrected)		(Voxels)
**ADHD > TDCs**							
No cluster found							
**ADHD < TDCs** Left crus II (Cerebellum)	−22	−78	−36	−3.569	0.02	120	Left crus II (73) Left crus I (44) Left lobule VIIB (3)
Left crus I (Cerebellum)	−32	−58	−44	−3.525	0.03	84	Left crus II (51) Left crus I (11) Left lobule VIIB (10) Left lobule VI (9) Left lobule VII (2) Middle cerebellar peduncles (1)

Abbreviations: ADHD, attention-deficit/hyper-activity disorder; TDCs, typically developing controls; BA, Brodmann area; MNI, Montreal Neurological Institute; SDM, Seed-based d Mapping.

## Data Availability

Supplementary Materials accompany this paper. Meta-analytic data used in the present work are available as Supplementary Materials.

## Supplementary Materials

The following supporting information can be downloaded at: https://www.mdpi.com/article/10.3390/brainsci12101367/s1. Table S1. VBM experiments included in the original coordinate-based meta-analysis by McGrath and Stoodley (2019): methodological details for the attention-deficit/hyper-activity disorder (A) and dyslexia (B) datasets. Table S2. VBM experiments with null results and not included in the original coordinate-based meta-analysis by McGrath and Stoodley (2019): methodological details for the attention-deficit/hyper-activity disorder (A) and dyslexia (B) datasets. Table S3. Brain clusters of gray matter variation in attention-deficit/hyper-activity disorder compared with typically developmental controls at *p*_uncorrected_ < 0.0005 and minimum cluster size = 10 voxels (replication analysis). Table S4. Brain clusters of gray matter variation in attention-deficit/hyper-activity disorder compared with typically developmental controls at *p*_uncorrected_ < 0.0005 and minimum cluster size = 10 voxels (replication analysis). Table S5. Brain clusters of gray matter variation in attention-deficit/hyper-activity disorder compared with typically developmental controls at *p*_uncorrected_ < 0.0005 and minimum cluster size = 10 voxels (additional analysis). Table S6. Brain clusters of gray matter variation in dyslexia compared with typically developmental controls at *p*_uncorrected_ < 0.0005 and minimum cluster size = 10 voxels (replication analysis). Table S7. Brain clusters of gray matter variation in dyslexia compared with typically developmental controls at *p*_uncorrected_ < 0.0005 and minimum cluster size = 10 voxels (brain volume sub-analysis). Table S8. Brain clusters of gray matter variation in attention-deficit/hyper-activity disorder compared with typically developmental controls at *p*_uncorrected_ < 0.0005 and minimum cluster size = 10 voxels (brain volume sub-analysis). Figure S1. Brain clusters of gray matter variation in attention-deficit/hyper-activity disorder compared with typically developmental controls at *p*_uncorrected_ < 0.0005 and minimum cluster size = 10 voxels (replication analysis). Figure S2. Brain clusters of gray matter variation in attention-deficit/hyper-activity disorder compared with typically developmental controls at *p*_uncorrected_ < 0.0005 and minimum cluster size = 10 voxels (additional analysis). Figure S3. Brain clusters of gray matter variation in dyslexia compared with typically developmental controls at *p*_uncorrected_ < 0.0005 and minimum cluster size = 10 voxels (brain volume sub-analysis). Figure S4. Brain clusters of gray matter variation in attention-deficit/hyper-activity disorder compared with typically developmental controls at *p*_uncorrected_ < 0.0005 and minimum cluster size = 10 voxels (brain volume sub-analysis).

## References

[1] McGrath L.M., Stoodley C.J. (2019). Are there shared neural correlates between dyslexia and ADHD? A meta-analysis of voxel-based morphometry studies. J. Neurodev. Disord..

[2] Sánchez-Morán M., Hernández J.A., Duñabeitia J.A., Estévez A., Bárcena L., González-Lahera A., Bajo M.T., Fuentes L.J., Aransay A.M., Carreiras M. (2018). Genetic association study of dyslexia and ADHD candidate genes in a Spanish cohort: Implications of comorbid samples. PLoS ONE.

[3] Carlsson T., Molander F., Taylor M.J., Jonsson U., Bölte S. (2020). Early environmental risk factors for neurodevelopmental disorders—A systematic review of twin and sibling studies. Dev. Psychopathol..

[4] Micai M., Fulceri F., Caruso A., Guzzetta A., Gila L., Scattoni M.L. (2020). Early behavioral markers for neurodevelopmental disorders in the first 3 years of life: An overview of systematic reviews. Neurosci. Biobehav. Rev..

[5] Parenti I., Rabaneda L.G., Schoen H., Novarino G. (2020). Neurodevelopmental Disorders: From Genetics to Functional Pathways. Trends Neurosci..

[6] Battaglia S., Cardellicchio P., Di Fazio C., Nazzi C., Fracasso A., Borgomaneri S. (2022). The Influence of Vicarious Fear-Learning in “Infecting” Reactive Action Inhibition. Front. Behav. Neurosci..

[7] Tanaka M., Spekker E., Szabó Á., Polyák H., Vécsei L. (2022). Modelling the neurodevelopmental pathogenesis in neuropsychiatric disorders. Bioactive kynurenines and their analogues as neuroprotective agents-in celebration of 80th birthday of Professor Peter Riederer. J. Neural. Transm..

[8] Tanaka M., Tóth F., Polyák H., Szabó Á., Mándi Y., Vécsei L. (2021). Immune Influencers in Action: Metabolites and Enzymes of the Tryptophan-Kynurenine Metabolic Pathway. Biomedicines.

[9] Salem H.A., Elsherbiny N., Alzahrani S., Alshareef H.M., Abd Elmageed Z.Y., Ajwah S.M., Hamdan A.M.E., Abdou Y.S., Galal O.O., El Azazy M.K.A. (2022). Neuroprotective Effect of Morin Hydrate against Attention-Deficit/Hyperactivity Disorder (ADHD) Induced by MSG and/or Protein Malnutrition in Rat Pups: Effect on Oxidative/Monoamines/Inflammatory Balance and Apoptosis. Pharmaceuticals.

[10] Bush G., Valera E.M., Seidman L.J. (2005). Functional neuroimaging of attention-deficit/hyperactivity disorder: A review and suggested future directions. Biol. Psych..

[11] Cubillo A., Halari R., Smith A., Taylor E., Rubia K. (2012). A review of fronto-striatal and fronto-cortical brain abnormalities in children and adults with Attention Deficit Hyperactivity Disorder (ADHD) and new evidence for dysfunction in adults with ADHD during motivation and attention. Cortex.

[12] Sebastian A., Jung P., Krause-Utz A., Lieb K., Schmahl C., Tüscher O. (2014). Frontal dysfunctions of impulse control—A systematic review in borderline personality disorder and attention-deficit/hyperactivity disorder. Front. Hum. Neurosci..

[13] Rubia K. (2018). Cognitive Neuroscience of Attention Deficit Hyperactivity Disorder (ADHD) and Its Clinical Translation. Front. Hum. Neurosci..

[14] Damiani S., Tarchi L., Scalabrini A., Marini S., Provenzani U., Rocchetti M., Oliva F., Politi P. (2021). Beneath the surface: Hyper-connectivity between caudate and salience regions in ADHD fMRI at rest. Eur. Child. Adolesc. Psychiatry.

[15] American Psychiatric Association (2013). Diagnostic and Statistical Manual of Mental Disorders, Fth Edition (DSM-5).

[16] Maisog J.M., Einbinder E.R., Flowers D.L., Turkeltaub P.E., Eden G.F. (2008). A meta-analysis of functional neuroimaging studies of dyslexia. Ann. N. Y. Acad. Sci..

[17] Richlan F., Kronbichler M., Wimmer H. (2009). Functional abnormalities in the dyslexic brain: A quantitative meta-analysis of neuroimaging studies. Hum. Brain Mapp..

[18] Paulesu E., Danelli L., Berlingeri M. (2014). Reading the dyslexic brain: Multiple dysfunctional routes revealed by a new meta-analysis of PET and fMRI activation studies. Front. Hum. Neurosci..

[19] Elnakib A., Soliman A., Nitzken M., Casanova M.F., Gimel’farb G., El-Baz A. (2014). Magnetic resonance imaging findings for dyslexia: A review. J. Biomed. Nanotechnol..

[20] Eckert M. (2004). Neuroanatomical markers for dyslexia: A review of dyslexia structural imaging studies. Neuroscientist.

[21] Kibby M.Y., Kroese J.M., Krebbs H., Hill C.E., Hynd G.W. (2009). The pars triangularis in dyslexia and ADHD: A comprehensive approach. Brain Lang..

[22] Goradia D.D., Vogel S., Mohl B., Khatib D., Zajac-Benitez C., Rajan U., Robin A., Rosenberg D.R., Stanley J.A. (2016). Distinct differences in striatal dysmorphology between attention deficit hyperactivity disorder boys with and without a comorbid reading disability. Psychiatry Res. Neuroimag..

[23] Jagger-Rickels A.C., Kibby M.Y., Constance J.M. (2018). Global gray matter morphometry differences between children with reading disability, ADHD, and comorbid reading disability/ADHD. Brain Lang.

[24] Langer N., Benjamin C., Becker B.L.C., Gaab N. (2019). Comorbidity of reading disabilities and ADHD: Structural and functional brain characteristics. Hum. Brain Mapp..

[25] Ashburner J., Friston K.J. (2000). Voxel-based morphometry--the methods. NeuroImage.

[26] Caspers J., Zilles K., Beierle C., Rottschy C., Eickhoff S.B. (2014). A novel meta-analytic approach: Mining frequent co-activation patterns in neuroimaging databases. NeuroImage.

[27] Salimi-Khorshidi G., Smith S.M., Keltner J.R., Wager T.D., Nichols T.E. (2009). Meta-analysis of neuroimaging data: A comparison of image-based and coordinate-based pooling of studies. NeuroImage.

[28] Samartsidis P., Montagna S., Nichols T.E., Johnson T.D. (2017). The coordinate-based meta-analysis of neuroimaging data. Stat. Sci..

[29] Smith S.M., Beckmann C.F., Ramnani N., Woolrich M.W., Bannister P.R., Jenkinson M., Matthews P.M., McGonigle D.J. (2005). Variability in fMRI: A re-examination of inter-session differences. Hum. Brain Mapp..

[30] Manuello J., Costa T., Cauda F., Liloia D. (2022). Six actions to improve detection of critical features for neuroimaging coordinate-based meta-analysis preparation. Neurosci. Biobehav. Rev..

[31] Müller V.I., Cieslik E.C., Laird A.R., Fox P.T., Radua J., Mataix-Cols D., Tench C.R., Yarkoni T., Nichols T.E., Turkeltaub P.E. (2018). Ten simple rules for neuroimaging meta-analysis. Neurosci. Biobehav. Rev..

[32] Eickhoff S.B., Laird A.R., Grefkes C., Wang L.E., Zilles K., Fox P.T. (2009). Coordinate-based activation likelihood estimation meta-analysis of neuroimaging data: A random-effects approach based on empirical estimates of spatial uncertainty. Hum. Brain Mapp..

[33] Eickhoff S.B., Nichols T.E., Laird A.R., Hoffstaedter F., Amunts K., Fox P.T., Bzdok D., Eickhoff C.R. (2016). Behavior, sensitivity, and power of activation likelihood estimation characterized by massive empirical simulation. Neuroimage.

[34] Turkeltaub P.E., Eden G.F., Jones K.M., Zeffiro T.A. (2002). Meta-analysis of the functional neuroanatomy of single-word reading: Method and validation. Neuroimage.

[35] Nani A., Manuello J., Mancuso L., Liloia D., Costa T., Vercelli A., Duca S., Cauda F. (2021). The pathoconnectivity network analysis of the insular cortex: A morphometric fingerprinting. NeuroImage.

[36] Mancuso L., Fornito A., Costa T., Ficco L., Liloia D., Manuello J., Duca S., Cauda F. (2020). A meta-analytic approach to mapping co-occurrent grey matter volume increases and decreases in psychiatric disorders. NeuroImage.

[37] Eickhoff S.B., Bzdok D., Laird A.R., Roski C., Caspers S., Zilles K., Fox P.T. (2011). Co-activation patterns distinguish cortical modules, their connectivity and functional differentiation. NeuroImage.

[38] Albajes-Eizagirre A., Solanes A., Vieta E., Radua J. (2019). Voxel-based meta-analysis via permutation of subject images (PSI): Theory and implementation for SDM. NeuroImage.

[39] Stoodley C.J. (2014). Distinct regions of the cerebellum show gray matter decreases in autism, ADHD, and developmental dyslexia. Front. Syst. Neurosci..

[40] Samea F., Soluki S., Nejati V., Zarei M., Cortese S., Eickhoff S.B., Tahmasian M., Eickhoff C.R. (2019). Brain alterations in children/adolescents with ADHD revisited: A neuroimaging meta-analysis of 96 structural and functional studies. Neurosci. Biobehav. Rev..

[41] Radua J., Mataix-Cols D., Phillips M.L., El-Hage W., Kronhaus D.M., Cardoner N., Surguladze S. (2012). A new meta-analytic method for neuroimaging studies that combines reported peak coordinates and statistical parametric maps. Eur. Psychiatry.

[42] Ahrendts J., Rüsch N., Wilke M., Philipsen A., Eickhoff S.B., Glauche V., Perlov E., Ebert D., Hennig J., Tebartz Van Elst L. (2011). Visual Cortex Abnormalities in Adults with ADHD: A Structural MRI Study. World J. Biol. Psychiatry.

[43] Bonath B., Tegelbeckers J., Wilke M., Flechtner H.H., Krauel K. (2018). Regional Gray Matter Volume Differences Between Adolescents With ADHD and Typically Developing Controls: Further Evidence for Anterior Cingulate Involvement. J. Atten. Disord..

[44] Bralten J., Greven C.U., Franke B., Mennes M., Zwiers M.P., Rommelse N.N.J., Hartman C., van der Meer D., O’Dwyer L., Oosterlaan J. (2016). Voxel-Based Morphometry Analysis Reveals Frontal Brain Differences in Participants with ADHD and Their Unaffected Siblings. J. Psychiatry Neurosci..

[45] Brieber S., Neufang S., Bruning N., Kamp-Becker I., Remschmidt H., Herpertz-Dahlmann B., Fink G.R., Konrad K. (2007). Structural Brain Abnormalities in Adolescents with Autism Spectrum Disorder and Patients with Attention Deficit/Hyperactivity Disorder. J. Child Psychol. Psychiatry Allied Discip..

[46] Carmona S., Vilarroya O., Bielsa A., Trèmols V., Soliva J.C., Rovira M., Tomàs J., Raheb C., Gispert J.D., Batlle S. (2005). Global and Regional Gray Matter Reductions in ADHD: A Voxel-Based Morphometric Study. Neurosci. Lett..

[47] He N., Li F., Li Y., Guo L., Chen L., Huang X., Lui S., Gong Q. (2015). Neuroanatomical Deficits Correlate with Executive Dysfunction in Boys with Attention Deficit Hyperactivity Disorder. Neurosci. Lett..

[48] Iannaccone R., Hauser T.U., Ball J., Brandeis D., Walitza S., Brem S. (2015). Classifying Adolescent Attention-Deficit/Hyperactivity Disorder (ADHD) Based on Functional and Structural Imaging. Eur. Child Adolesc. Psychiatry.

[49] Johnston B.A., Mwangi B., Matthews K., Coghill D., Konrad K., Steele J.D. (2014). Brainstem Abnormalities in Attention Deficit Hyperactivity Disorder Support High Accuracy Individual Diagnostic Classification. Hum. Brain Mapp..

[50] Kappel V., Lorenz R.C., Streifling M., Renneberg B., Lehmkuhl U., Ströhle A., Salbach-Andrae H., Beck A. (2015). Effect of Brain Structure and Function on Reward Anticipation in Children and Adults with Attention Deficit Hyperactivity Disorder Combined Subtype. Soc. Cogn. Affect. Neurosci..

[51] Sutcubasi Kaya B., Metin B., Tas Z.C., Buyukaslan A., Soysal A., Hatiloglu D., Tarhan N. (2018). Gray Matter Increase in Motor Cortex in Pediatric ADHD: A Voxel-Based Morphometry Study. J. Atten. Disord..

[52] Kobel M., Bechtel N., Specht K., Klarhöfer M., Weber P., Scheffler K., Opwis K., Penner I.K. (2010). Structural and Functional Imaging Approaches in Attention Deficit/Hyperactivity Disorder: Does the Temporal Lobe Play a Key Role?. Psychiatry Res. Neuroimag..

[53] Kumar U., Arya A., Agarwal V. (2017). Neural Alterations in ADHD Children as Indicated by Voxel-Based Cortical Thickness and Morphometry Analysis. Brain Dev..

[54] Lim L., Marquand A., Cubillo A.A., Smith A.B., Chantiluke K., Simmons A., Mehta M., Rubia K. (2013). Disorder-Specific Predictive Classification of Adolescents with Attention Deficit Hyperactivity Disorder (ADHD) Relative to Autism Using Structural Magnetic Resonance Imaging. PLoS ONE.

[55] McAlonan G.M., Cheung V., Cheung C., Chua S.E., Murphy D.G.M., Suckling J., Tai K.S., Yip L.K.C., Leung P., Ho T.P. (2007). Mapping Brain Structure in Attention Deficit-Hyperactivity Disorder: A Voxel-Based MRI Study of Regional Grey and White Matter Volume. Psychiatry Res. Neuroimag..

[56] Montes L.G.A., Ricardo-Garcell J., de la Torre L.B., Alcántara H.P., García R.B.M., Fernández-Bouzas A., Acosta D.Á. (2010). Clinical Correlations of Grey Matter Reductions in the Caudate Nucleus of Adults with Attention Deficit Hyperactivity Disorder. J. Psychiatry Neurosci..

[57] Moreno-Alcázar A., Ramos-Quiroga J.A., Radua J., Salavert J., Palomar G., Bosch R., Salvador R., Blanch J., Casas M., McKenna P.J. (2016). Brain Abnormalities in Adults with Attention Deficit Hyperactivity Disorder Revealed by Voxel-Based Morphometry. Psychiatry Res. Neuroimag..

[58] Overmeyer S., Bullmore E.T., Suckling J., Simmons A., Williams S.C.R., Santosh P.J., Taylor E. (2001). Distributed Grey and White Matter Deficits in Hyperkinetic Disorder: MRI Evidence for Anatomical Abnormality in an Attentional Network. Psychol. Med..

[59] Roman-Urrestarazu A., Lindholm P., Moilanen I., Kiviniemi V., Miettunen J., Jääskeläinen E., Mäki P., Hurtig T., Ebeling H., Barnett J.H. (2016). Brain Structural Deficits and Working Memory FMRI Dysfunction in Young Adults Who Were Diagnosed with ADHD in Adolescence. Eur. Child Adolesc. Psychiatry.

[60] Sasayama D., Hayashida A., Yamasue H., Harada Y., Kaneko T., Kasai K., Washizuka S., Amano N. (2010). Neuroanatomical Correlates of Attention-Deficit-Hyperactivity Disorder Accounting for Comorbid Oppositional Defiant Disorder and Conduct Disorder. Psychiatry Clin. Neurosci..

[61] van Wingen G.A., van den Brink W., Veltman D.J., Schmaal L., Dom G., Booij J., Crunelle C.L. (2013). Reduced Striatal Brain Volumes in Non-Medicated Adult ADHD Patients with Comorbid Cocaine Dependence. Drug Alcohol. Depend..

[62] Villemonteix T., De Brito S.A., Kavec M., Balériaux D., Metens T., Slama H., Baijot S., Mary A., Peigneux P., Massat I. (2015). Grey Matter Volumes in Treatment Naïve vs. Chronically Treated Children with Attention Deficit/Hyperactivity Disorder: A Combined Approach. Eur. Neuropsychopharmacol..

[63] Yang P., Wang P.N., Chuang K.H., Jong Y.J., Chao T.C., Wu M.T. (2008). Absence of Gender Effect on Children with Attention-Deficit/Hyperactivity Disorder as Assessed by Optimized Voxel-Based Morphometry. Psychiatry Res. Neuroimag..

[64] Brambati S.M., Termine C., Ruffino M., Stella G., Fazio F., Cappa S.F., Perani D. (2004). Regional Reductions of Gray Matter Volume in Familial Dyslexia. Neurology.

[65] Brown W.E., Eliez S., Menon V., Rumsey J.M., White C.D., Reiss A.L. (2001). Preliminary Evidence of Widespread Morphological Variations of the Brain in Dyslexia. Neurology.

[66] Eckert M.A., Leonard C.M., Wilke M., Eckert M., Richards T., Richards A., Berninger V. (2005). Anatomical Signatures of Dyslexia in Children: Unique Information from Manual and Voxel Based Morphometry Brain Measures. Cortex.

[67] Evans T.M., Flowers D.L., Napoliello E.M., Eden G.F. (2014). Sex-Specific Gray Matter Volume Differences in Females with Developmental Dyslexia. Brain Struct. Funct..

[68] Hoeft F., Meyler A., Hernandez A., Juel C., Taylor-Hill H., Martindale J.L., McMillon G., Kolchugina G., Black J.M., Faizi A. (2007). Functional and Morphometric Brain Dissociation between Dyslexia and Reading Ability. Proc. Natl. Acad. Sci. USA.

[69] Jednoróg K., Marchewka A., Altarelli I., Monzalvo Lopez A.K., van Ermingen-Marbach M., Grande M., Grabowska A., Heim S., Ramus F. (2015). How Reliable Are Gray Matter Disruptions in Specific Reading Disability across Multiple Countries and Languages? Insights from a Large-Scale Voxel-Based Morphometry Study. Hum. Brain Mapp..

[70] Kronbichler M., Wimmer H., Staffen W., Hutzier F., Mair A., Ladurner G. (2008). Developmental Dyslexia: Gray Matter Abnormalities in the Occipitotemporal Cortex. Hum. Brain Mapp..

[71] Liu L., You W., Wang W., Guo X., Peng D., Booth J. (2013). Altered Brain Structure in Chinese Dyslexic Children. Neuropsychologia.

[72] Silani G., Frith U., Demonet J.F., Fazio F., Perani D., Price C., Frith C.D., Paulesu E. (2005). Brain Abnormalities Underlying Altered Activation in Dyslexia: A Voxel Based Morphometry Study. Brain.

[73] Wai T.S., Niu Z., Jin Z., Perfetti C.A., Li H.T. (2008). A Structural-Functional Basis for Dyslexia in the Cortex of Chinese Readers. Proc. Natl. Acad. Sci. USA.

[74] Steinbrink C., Vogt K., Kastrup A., Müller H.P., Juengling F.D., Kassubek J., Riecker A. (2008). The Contribution of White and Gray Matter Differences to Developmental Dyslexia: Insights from DTI and VBM at 3.0 T. Neuropsychologia.

[75] Tamboer P., Scholte H.S., Vorst H.C.M. (2015). Dyslexia and Voxel-Based Morphometry: Correlations between Five Behavioural Measures of Dyslexia and Gray and White Matter Volumes. Ann. Dyslexia.

[76] Vinckenbosch E., Robichon F., Eliez S. (2005). Gray Matter Alteration in Dyslexia: Converging Evidence from Volumetric and Voxel-by-Voxel MRI Analyses. Neuropsychologia.

[77] Xia Z., Hoeft F., Zhang L., Shu H. (2016). Neuroanatomical Anomalies of Dyslexia: Disambiguating the Effects of Disorder, Performance, and Maturation. Neuropsychologia.

[78] Yang Y.H., Yang Y., Chen B.G., Zhang Y.W., Bi H.Y. (2016). Anomalous Cerebellar Anatomy in Chinese Children with Dyslexia. Front. Psychol..

[79] Winkler A.M., Ridgway G.R., Webster M.A., Smith S.M., Nichols T.E. (2014). Permutation inference for the general linear model. NeuroImage.

[80] Radua J., Romeo M., Mataix-Cols D., Fusar-Poli P. (2013). A general approach for combining voxel-based meta-analyses conducted in different neuroimaging modalities. Curr. MedChem..

[81] Acar F., Seurinck R., Eickhoff S.B., Moerkerke B. (2018). Assessing robustness against potential publication bias in Activation Likelihood Estimation (ALE) meta-analyses for fMRI. PLoS ONE.

[82] Laitin D.D., Miguel E., Alrababa’h A., Bogdanoski A., Grant S., Hoeberling K., Hyunjung Mo C., Moore D.A., Vazire S., Weinstein J. (2021). Reporting all results efficiently: A RARE proposal to open up the file drawer. Proc. Natl. Acad. Sci. USA.

[83] Amico F., Stauber J., Koutsouleris N., Frodl T. (2011). Anterior Cingulate Cortex Gray Matter Abnormalities in Adults with Attention Deficit Hyperactivity Disorder: A Voxel-Based Morphometry Study. Psychiatry Res. Neuroimag..

[84] Depue B.E., Burgess G.C., Bidwell L.C., Willcutt E.G., Banich M.T. (2010). Behavioral Performance Predicts Grey Matter Reductions in the Right Inferior Frontal Gyrus in Young Adults with Combined Type ADHD. Psychiatry Res. Neuroimag..

[85] Maier S., Perlov E., Graf E., Dieter E., Sobanski E., Rump M., Warnke A., Ebert D., Berger M., Matthies S. (2016). Discrete Global but No Focal Gray Matter Volume Reductions in Unmedicated Adult Patients With Attention-Deficit/Hyperactivity Disorder. Biol. Psychiatry.

[86] Onnink A.M.H., Zwiers M.P., Hoogman M., Mostert J.C., Kan C.C., Buitelaar J., Franke B. (2014). Brain Alterations in Adult ADHD: Effects of Gender, Treatment and Comorbid Depression. Eur. Neuropsychopharmacol..

[87] Saad J.F., Griffiths K.R., Kohn M.R., Clarke S., Williams L.M., Korgaonkar M.S. (2017). Regional Brain Network Organization Distinguishes the Combined and Inattentive Subtypes of Attention Deficit Hyperactivity Disorder. NeuroImage Clin..

[88] Seidman L.J., Biederman J., Liang L., Valera E.M., Monuteaux M.C., Brown A., Kaiser J., Spencer T., Faraone S.V., Makris N. (2011). Gray Matter Alterations in Adults with Attention-Deficit/Hyperactivity Disorder Identified by Voxel Based Morphometry. Biol. Psychiatry.

[89] Villemonteix T., De Brito S.A., Slama H., Kavec M., Balériaux D., Metens T., Baijot S., Mary A., Peigneux P., Massat I. (2015). Grey Matter Volume Differences Associated with Gender in Children with Attention-Deficit/Hyperactivity Disorder: A Voxel-Based Morphometry Study. Dev. Cogn. Neurosci..

[90] Eckert M.A., Berninger V.W., Vaden K.I., Gebregziabher M., Tsu L. (2016). Gray Matter Features of Reading Disability: A Combined Meta-Analytic and Direct Analysis Approach. eNeuro.

[91] Pernet C., Andersson J., Paulesu E., Demonet J.F. (2009). When All Hypotheses Are Right: A Multifocal Account of Dyslexia. Hum. Brain Mapp..

[92] Laird A.R., Fox P.M., Price C.J., Glahn D.C., Uecker A.M., Lancaster J.L., Turkeltaub P.E., Kochunov P., Fox P.T. (2005). ALE meta-analysis: Controlling the false discovery rate and performing statistical contrasts. Hum. Brain Mapp..

[93] Eickhoff S.B., Laird A.R., Fox P.M., Lancaster J.L., Fox P.T. (2017). Implementation errors in the GingerALE Software: Description and recommendations. Hum. Brain Mapp..

[94] Tahmasian M., Sepehry A.A., Samea F., Khodadadifar T., Soltaninejad Z., Javaheripour N., Khazaie H., Zarei M., Eickhoff S.B., Eickhoff C.R. (2019). Practical recommendations to conduct a neuroimaging meta-analysis for neuropsychiatric disorders. Hum. Brain Mapp..

[95] Liloia D., Brasso C., Cauda F., Mancuso L., Nani A., Manuello J., Costa T., Duca S., Rocca P. (2021). Updating and characterizing neuroanatomical markers in high-risk subjects, recently diagnosed and chronic patients with schizophrenia: A revised coordinate-based meta-analysis. Neurosci. Biobehav. Rev..

[96] Mostofsky S.H., Reiss A.L., Lockhart P., Denckla M.B. (1998). Evaluation of cerebellar size in attention-deficit hyperactivity disorder. J. Child. Neurol..

[97] Goetz M., Vesela M., Ptacek R. (2014). Notes on the role of the cerebellum in ADHD. Austin. J. Psychiatry Behav. Sci..

[98] Stoodley C.J. (2016). The Cerebellum and Neurodevelopmental Disorders. Cerebellum.

[99] Bruchhage M.M.K., Bucci M.P., Becker E.B.E. (2018). Cerebellar involvement in autism and ADHD. Handd. Clin. Neurol..

[100] Linkersdörfer J., Lonnemann J., Lindberg S., Hasselhorn M., Fiebach C.J. (2012). Grey matter alterations co-localize with functional abnormalities in developmental dyslexia: An ALE meta-analysis. PLoS ONE.

[101] Richlan F., Kronbichler M., Wimmer H. (2013). Structural abnormalities in the dyslexic brain: A meta-analysis of voxel-based morphometry studies. Hum. Brain Mapp..

[102] Yan X., Jiang K., Li H., Wang Z., Perkins K., Cao F. (2021). Convergent and divergent brain structural and functional abnormalities associated with developmental dyslexia. eLife.

[103] Albajes-Eizagirre A., Radua J. (2018). What do results from coordinate-based meta-analyses tell us?. NeuroImage.

[104] Shaywitz S.E., Escobar M.D., Shaywitz B.A., Fletcher J.M., Makuch R. (1992). Evidence That Dyslexia May Represent the Lower Tail of a Normal Distribution of Reading Ability. NEJM.

[105] Peterson R.L., Pennington B.F., Olson R.K. (2013). Subtypes of developmental dyslexia: Testing the predictions of the dual-route and connectionist frameworks. Cognition.

[106] Kern J.K., Geier D.A., Sykes L.K., Geier M.R., Deth R.C. (2015). Are ASD and ADHD a Continuum? A Comparison of Pathophysiological Similarities Between the Disorders. JAD.

[107] Whitely M. (2015). Attention deficit hyperactive disorder diagnosis continues to fail the reliability and validity tests. Aust. N. Z. J. Psychiatry.

[108] McLennan J.D. (2016). Understanding attention deficit hyperactivity disorder as a continuum. Can. Fam. Physician Med. Fam. Can..

[109] Gnanavel S., Sharma P., Kaushal P., Hussain S. (2019). Attention deficit hyperactivity disorder and comorbidity: A review of literature. World J. Clin. Cases.

[110] Darweesh A.M., Elserogy Y.M., Khalifa H., Gabra R.H., El-Ghafour M.A. (2020). Psychiatric comorbidity among children and adolescents with dyslexia. MECP.

