# Supplementary material for: Seeking Overlapping Neuroanatomical Alterations between Dyslexia and Attention-Deficit/Hyperactivity Disorder: A Meta-Analytic Replication Study
Source: Brain Sci. 2022 Oct 9;12(10):1367. doi: 10.3390/brainsci12101367 (PMC9599506; doi:10.3390/brainsci12101367)
Supplement: Supplementary file 1 [file brainsci-12-01367-s001.zip › Supplementary_Materials_Data/Supplementary_Materials_Revised.pdf]

Are there shared neural correlates between dyslexia and ADHD?
A meta-analysis of voxel-based morphometry studies – Open-Data Replication

Supplementary Material

DATASET CONSTRUCTION

Table S1. VBM experiments included in the original coordinate-based meta-analysis by McGrath and Stoodley (2019): methodological details for the attention-deficit/hyper-activity disorder (A) and dyslexia (B) datasets …***page 3***

Table S2. VBM experiments with null results and not included in the original coordinate-based meta-analysis by McGrath and Stoodley (2019): methodological details for the attention-deficit/hyper-activity disorder (A) and dyslexia (B) datasets …***page 6***

REPLICATION ANALYSIS

Table S3. Brain clusters of gray matter variation in attention-deficit/hyper-activity disorder compared with typically developmental controls at $p_{uncorrected}$ <0.0005 and minimum cluster size = 10 voxels (replication analysis) …***page 7***

Figure S1. Brain clusters of gray matter variation in attention-deficit/hyper-activity disorder compared with typically developmental controls at $p_{uncorrected}$ <0.0005 and minimum cluster size = 10 voxels (replication analysis) …***page 8***

Table S4. Brain clusters of gray matter variation in dyslexia compared with typically developmental controls at $p_{uncorrected}$ <0.0005 and minimum cluster size = 10 voxels (replication analysis) …***page 9***

ADDITIONAL ANALYSIS

Table S5. Brain clusters of gray matter variation in attention-deficit/hyper-activity disorder compared with typically developmental controls at $p_{uncorrected}$ <0.0005 and minimum cluster size = 10 voxels (additional analysis) …***page 10***

Figure S2. Brain clusters of gray matter variation in attention-deficit/hyper-activity disorder compared with typically developmental controls at $p_{uncorrected}$ <0.0005 and minimum cluster size = 10 voxels (additional analysis) …***page 11***

Table S6. Brain clusters of gray matter variation in dyslexia compared with typically developmental controls at $p_{uncorrected}$ <0.0005 and minimum cluster size = 10 voxels (additional analysis) …***page 12***

BRAIN VOLUME SUB-ANALYSIS

Table S7. Brain clusters of gray matter variation in dyslexia compared with typically developmental controls at $p_{uncorrected}$ <0.0005 and minimum cluster size = 10 voxels (brain volume sub-analysis) …***page 13***

Figure S3. Brain clusters of gray matter variation in dyslexia compared with typically developmental controls at $p_{uncorrected}$ <0.0005 and minimum cluster size = 10 voxels (brain volume sub-analysis) …***page 14***

Table S8. Brain clusters of gray matter variation in attention-deficit/hyper-activity disorder compared with typically developmental controls at $p_{uncorrected}$ <0.0005 and minimum cluster size = 10 voxels (brain volume sub-analysis) …***page 15***

Figure S4. Brain clusters of gray matter variation in attention-deficit/hyper-activity disorder compared with typically developmental controls at $p_{uncorrected}$ <0.0005 and minimum cluster size = 10 voxels (brain volume sub-analysis) …***page 17***

References …page 18

DATASET CONSTRUCTION

Table S1. VBM experiments included in the original coordinate-based meta-analysis by McGrath and Stoodley (2019): methodological details for the attention-deficit/hyper-activity disorder (A) and dyslexia (B) datasets.

Experiments	GM variations TDCs > PZ	GM variations PZ > TDCs	P value (correction)	VBM software	Thickness (mm)	Smoothing (FWHM)	Scanner (Tesla)	Original data
**(A) ADHD**								
Ahrendts et al., 2011	2	0	p < 0.05 corrected for multiple comparisons using FDR	SPM 2	1	12 mm	1.5	MNI
Bonath et al., 2018	12	0	p < 0.001 uncorrected on the voxel level<br>p < 0.05 corrected for multiple comparisons using FDR	SPM 8	1	8 mm	3.0	BRETT
Bralten et al., 2016	5	0	p < 0.05 corrected for multiple comparisons using FWE	SPM	N/A	8 mm	1.5	MNI
Brieber et al., 2007	9	6	p < 0.001 uncorrected for multiple comparisons	SPM 2	1	12 mm	1.5	MNI
Carmona et al., 2005	17	0	p < 0.05 corrected for multiple comparisons using FWE	SPM 2	N/A	12 mm	1.5	MNI
He et al., 2015	4	0	p < 0.05 corrected for multiple comparisons using FWE	SPM 8	1	8 mm	3.0	MNI
Iannaccone et al., 2015	3	2	p < 0.05 corrected for multiple comparisons using cluster extent correction	SPM 8	1	8 mm	3.0	MNI
Johnston et al., 2014	12	0	p < 0.05 corrected for multiple comparisons using FWE	SPM 8	1	8 mm	1.5	MNI
Kappel et al., 2015 (adults)	4	0	p < 0.05 corrected for multiple comparisons using FWE	SPM 8	1	6 mm	3.0	MNI
Kappel et al., 2015 (children)	1	4	p < 0.05 corrected for multiple comparisons using FWE	SPM 8	1	6 mm	3.0	MNI
Kaya et al., 2018	0	7	p < 0.001 uncorrected for multiple comparisons	SPM 8	N/A	8 mm	1.5	MNI
Kobel et al. 2010	1	0	p < 0.001 uncorrected	SPM 5	N/A	12 mm	3.0	TAL
Kumar et al., 2017	4	0	p < 0.05 corrected for multiple comparisons using FDR	SPM 8	N/A	12 mm	3.0	OTHER
Lim et al., 2013	6	0	p < 0.05 corrected for multiple comparisons using FWE	SPM 8	N/A	8 mm	3.0	TAL
McAlonan et al., 2007	8	0	p < 0.001 uncorrected for volume differences<br>p < 0.05 corrected for multiple comparisons using FWE	BAMM	3	4.4 mm	1.5	TAL
Montes et al., 2010	2	0	p < 0.01 corrected for multiple comparisons using FDR and FWE	SPM 5	1	8 mm	1.0	MNI

Moreno-Alcázar et al., 2016	3	1	$p < 0.001$ uncorrected on the voxel level<br>$p < 0.05$ corrected for multiple comparisons using FWE	FSL	1	9.4 mm	1.5	MNI
Overmeyer et al., 2001	9	0	$p < 0.025$ uncorrected	N/A	3	N/A	1.5	TAL
Roman-Urrestarazu et al., 2016	2	0	$p < 0.05$ corrected for multiple comparisons using FWE	FSL-VBM	1	3 mm	1.5	MNI
Sasayama et al., 2010	14	0	$p < 0.05$ corrected for multiple comparisons using FDR	SPM 2	1	12 mm	1.5	MNI
van Wingen et al., 2013	2	2	$p < 0.05$ corrected for multiple comparisons using FWE	SPM 8	1.2	8 mm	3.0	MNI
Villemonteix et al., 2015 (med naïve group)	2	0	$p < 0.001$ uncorrected for multiple comparisons	SPM 8	N/A	5 mm	3.0	MNI
Villemonteix et al., 2015 (med group)	2	0	$p < 0.001$ uncorrected for multiple comparisons	SPM 8	N/A	5 mm	3.0	MNI
Yang et al., 2008	6	0	$p < 0.001$ uncorrected for multiple comparisons<br>$p < 0.05$ corrected for multiple comparisons	SPM 2	5	8 mm	1.5	MNI
**(B) DYSLEXIA**								
Brambati et al., 2004	9	0	$p < 0.05$ corrected for small brain volume	SPM 2	1.5	12 mm	1.5	TAL
Brown et al., 2001	8	0	$p < 0.05$ corrected for multiple comparisons	SPM 99	2	8 mm	1.5	TAL
Eckert et al., 2005	5	1	$p < 0.001$ uncorrected significance levels	SPM2b	N/A	12 mm	1.5	MNI
Evans et al., 2014 (male adults)	2	0	$p < 0.001$ uncorrected for statistical map generation<br>$p < 0.05$ extent threshold utilizing a non-stationary correction	SPM 8	1	8 mm	1.5/3.0	TAL
Evans et al., 2014 (female adults)	2	0	$p < 0.001$ uncorrected for statistical map generation<br>$p < 0.05$ extent threshold utilizing a non-stationary correction	SPM 8	1	8 mm	1.5/3.0	TAL
Evans et al., 2014 (male children)	1	0	$p < 0.001$ uncorrected for statistical map generation<br>$p < 0.05$ extent threshold utilizing a non-stationary correction	SPM 8	1	8 mm	1.5/3.0	TAL
Evans et al., 2014 (female children)	3	0	$p < 0.001$ uncorrected for statistical map generation<br>$p < 0.05$ extent threshold utilizing a non-stationary correction	SPM 8	1	8 mm	1.5/3.0	TAL
Hoeft et al., 2007	6	0	$p < 0.01$ corrected for whole brain analysis	SPM 2	N/A	8 mm	3.0	TAL
Jednoróg et al., 2015	1	0	$p < 0.05$ corrected for multiple comparisons	SPM 8	1	4 mm	1.5/3.0	MNI
Kronbichler et al., 2008	11	8	$p < 0.05$ corrected for multiple comparisons using FDR	SPM 2	1.3	12mm	1.5	MNI
Liu et al., 2013	8	0	$p < 0.001$ with clusters corrected for nonisotropic smoothness	SPM 5	1	12 mm	3.0	MNI
Silani et al., 2005	1	1	$p < 0.05$ corrected for whole brain	SPM 2	1.5	12 mm	1.5/2.0	TAL

Siok et al., 2008	3	0	$p < 0.05$ corrected for multiple comparisons using FWE	SPM 2	2	10 mm	2.0	MNI
Steinbrink et al., 2008	2	0	$p < 0.05$ corrected for multiple comparisons using FDR	SPM 5	N/A	12 mm	3.0	MNI
Tamboer et al., 2015	8	3	$p < 0.05$ corrected for multiple comparisons using random field theory	FSL-VBM	N/A	4 mm	3.0	MNI
Vinckenbosch et al., 2005	1	1	$p < 0.001$ uncorrected for multiple comparisons<br>$p < 0.05$ corrected for multiple comparisons using FDR and FWE	SPM99	1	8 mm	1.5	TAL
Xia et al., 2016	3	0	$p < 0.001$ uncorrected for multiple comparisons<br>$p < 0.05$ corrected for multiple comparisons using FWE	SPM8	1.33	8 mm	3.0	MNI
Yang et al., 2016	7	3	$p < 0.001$ corrected by Alpha Sim correction	SPM8	4	8 mm	1.5	MNI

ADHD, attention-deficit/hyper-activity disorder; FDR, false discovery rate; FWE, family-wise error; FWHM, full width at half maximum; MNI, Montreal Neurological Institute; N/A, data not available; PZ, patients; TAL, Talairach; TDCs, typically developing controls; VBM, voxel-based morphometry.

Table S2. VBM experiments with null results and not included in the original coordinate-based meta-analysis by McGrath and Stoodley (2019): methodological details for the attention-deficit/hyper-activity disorder (A) and dyslexia (B) datasets.

Experiments	P value (correction)	VBM software	Thickness (mm)	Smoothing (FWHM)	Scanner (Tesla)	Original data
**(A) ADHD**						
Amico et al., 2011	$p < 0.05$ corrected for multiple comparisons using FWE	SPM5	1.5	8 mm	1.5	TAL
Depue et al., 2010	$p < 0.05$ corrected for multiple comparisons	FSL-VBM	1.7	4.6 mm	N/A	MNI
Maier et al., 2016	$p < 0.05$ corrected for multiple comparisons using FWE	SPM12	N/A	8 mm	N/A	OTHER
Onnink et al., 2014	$p < 0.05$ corrected for multiple comparisons using FDR	SPM8	N/A	8 mm	1.5	MNI
Saad et al., 2017	$p < 0.05$ corrected for multiple comparisons using FDR	SPM8	1	N/A	3.0	MNI
Seidman et al., 2011	$p < 0.05$ corrected for multiple comparisons using FWE	FSL-VBM	1.33	7.05 mm	1.5	MNI
Villemonteix et al., 2015	$p < 0.05$ corrected for multiple comparisons using FWE	SPM8	N/A	12 mm	3.0	MNI
**(B) DYSLEXIA**						
Eckert et al., 2016	$p < 0.05$ corrected for multiple comparisons using FDR	SPM8	N/A	8 mm	1.5/3.0	MNI
Pernet et al., 2009	$p < 0.05$ corrected for multiple comparisons using FDR	SPM5	N/A	8 mm	1.5	MNI

ADHD, attention-deficit/hyper-activity disorder; FDR, false discovery rate; FWE, family-wise error; FWHM, full width at half maximum; MNI, Montreal Neurological Institute; N/A, data not available; PZ, patients; TAL, Talairach; TDCs, typically developing controls; VBM, voxel-based morphometry.

REPLICATION ANALYSIS

Table S3. Brain clusters of gray matter variation in attention-deficit/hyper-activity disorder compared with typically developmental controls at $p_{uncorrected}$ <0.0005 and minimum cluster size = 10 voxels (replication analysis).

Region	MNI coordinate			SDM	*P < 0.005*	Voxels	Cluster breakdown
	x	y	z	*Z* score	*(Uncorrected)*		(Voxels)
**ADHD > TDCs**							
No cluster found							
**ADHD < TDCs**							
Right superior frontal gyrus, medial orbital (BA 11)	6	24	-10	-3.708	0.0001	127	Right SFG (66)<br>Right gyrus rectus (32)<br>Right olfactory cortex (15)<br>Bilateral ACC (12)<br>Right Striatum (2)
Right lenticular nucleus (Putamen)	30	0	-2	-3.385	0.0003	82	Right Putamen (65)<br>Right Striatum (17)
Left lobule VI (Cerebellum)	-34	-46	-34	-2.783	0.002	22	Left lobule VI (16)<br>Left crus I (6)
Left postcentral gyrus (BA 6)	-46	-14	50	-2.942	0.001	12	Left PoCG (12)
Right gyrus rectus (BA 11)	4	38	-24	-2.781	0.002	10	Right gyrus rectus (10)

Abbreviations: ADHD, attention-deficit/hyper-activity disorder; TDCs, typically developing controls; BA, Brodmann area; MNI, Montreal Neurological Institute; SDM, Seed-based d Mapping; SFG, superior frontal gyrus; ACC, anterior cingulate cortex; PoCG, posterior central gyrus.

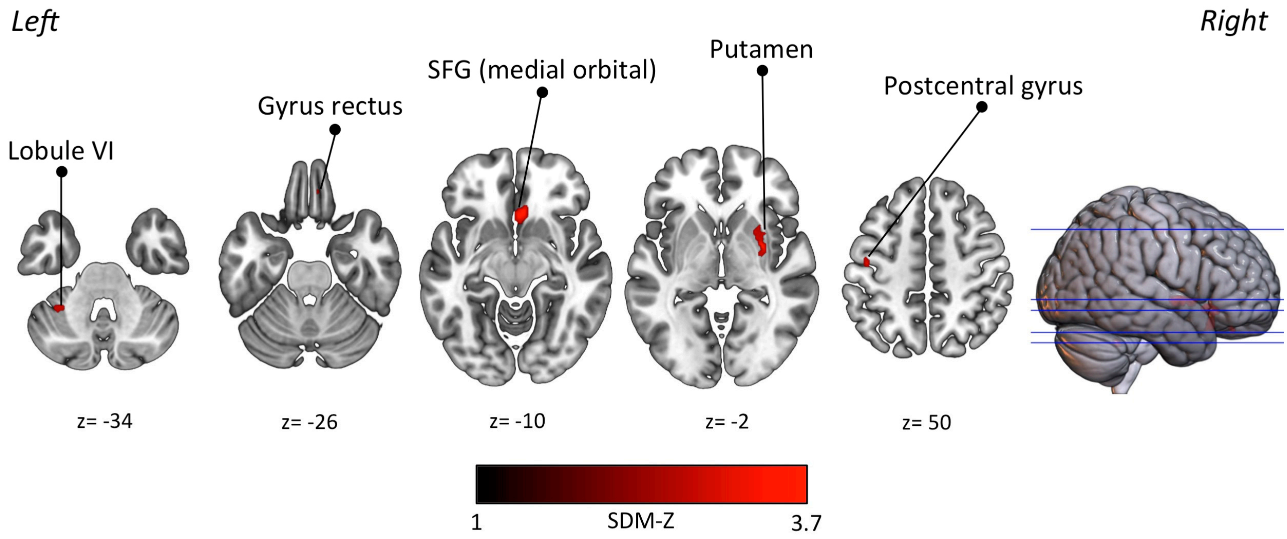

Figure S1. Brain clusters of gray matter variation in attention-deficit/hyper-activity disorder compared with typically developmental controls at $p_{uncorrected}$ <0.0005 and minimum cluster size = 10 voxels (replication analysis).

The PSI-SDM map is visualized as six axial slices (2-D cortical, subcortical, and cerebellar view). Colors from dark to light red represent voxels with a common pattern of neuroanatomical reduction (gray matter in attention-deficit/hyper-activity disorder < typically developmental controls). Brain slices are in neurological convention (i.e. Right is right, Left is left). SFG, superior frontal gyrus.

Table S4. Brain clusters of gray matter variation in attention-deficit/hyper-activity disorder compared with typically developmental controls at $p_{uncorrected}$ <0.0005 and minimum cluster size = 10 voxels (replication analysis).

**Region**	**MNI coordinate**			**SDM**	***P < 0.005***	**Voxels**	**Cluster breakdown**
	x	y	z	***Z* score**	***(Uncorrected)***		**(Voxels)**
**Dyslexia > TDCs**							
No cluster found							
**Dyslexia < TDCs**							
No cluster found							

Abbreviations: TDCs, typically developing controls; SDM, Seed-based d Mapping.

ADDITIONAL ANALYSIS

Table S5. Brain clusters of gray matter variation in attention-deficit/hyper-activity disorder compared with typically developmental controls at $p_{uncorrected}$ <0.0005 and minimum cluster size = 10 voxels (additional analysis).

Region	MNI coordinate			SDM	*P < 0.005*	Voxels	Cluster breakdown
	x	y	z	*Z* score	*(Uncorrected)*		(Voxels)
**ADHD > TDCs**							
No cluster found							
**ADHD < TDCs**							
Right superior frontal gyrus, medial orbital (BA 11)	6	24	-10	-4.051	0.00002	148	Right SFG (87)<br>Right gyrus rectus (38)<br>Right olfactory cortex (10)<br>Bilateral ACC (9)<br>Right Striatum (4)
Right lenticular nucleus (Putamen)	30	0	-2	-3.817	0.00006	152	Right Putamen (131)<br>Right Striatum (21)
Left lobule VI (Cerebellum)	-34	-46	-34	-3.023	0.001	50	Left lobule VI (29)<br>Left crus I (21)
Right gyrus rectus (BA 11)	4	38	-24	-3.281	0.0005	19	Right gyrus rectus (19)

Abbreviations: ADHD, attention-deficit/hyper-activity disorder; TDCs, typically developing controls; BA, Brodmann area; MNI, Montreal Neurological Institute; SDM, Seed-based d Mapping; SFG, superior frontal gyrus; ACC, anterior cingulate cortex.

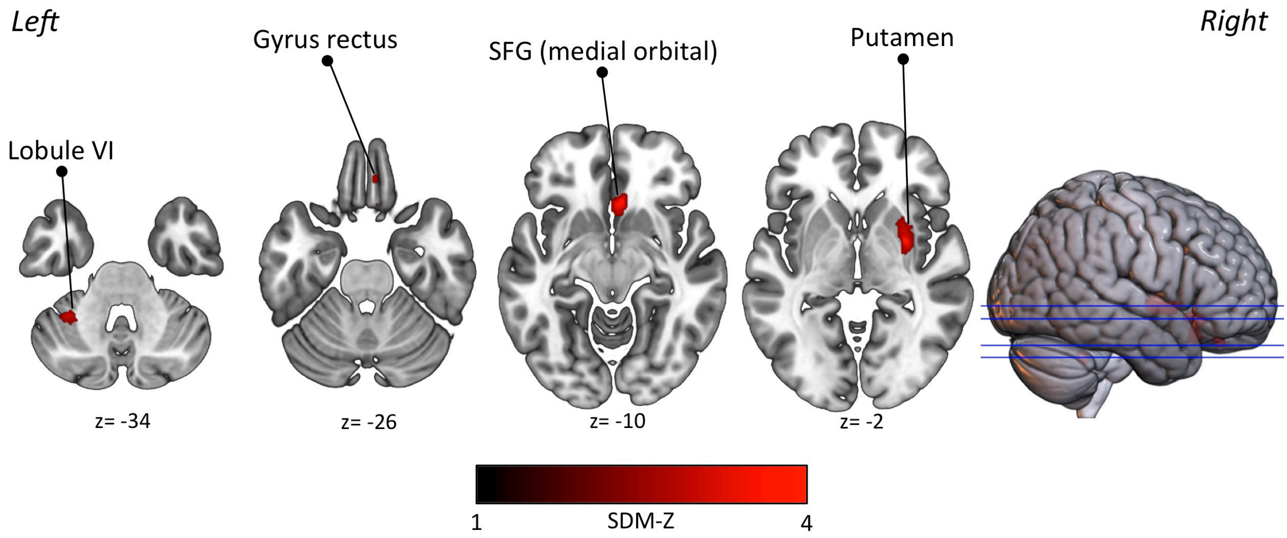

Figure S2. Brain clusters of gray matter variation in attention-deficit/hyper-activity disorder compared with typically developmental controls at $p_{uncorrected}$ <0.0005 and minimum cluster size = 10 voxels (additional analysis).

The PSI-SDM map is visualized as six axial slices (2-D cortical, subcortical, and cerebellar view). Colors from dark to light red represent voxels with a common pattern of neuroanatomical reduction (gray matter in attention-deficit/hyper-activity disorder < typically developmental controls). Brain slices are in neurological convention (i.e. Right is right, Left is left). SFG, superior frontal gyrus.

Table S6. Brain clusters of gray matter variation in dyslexia compared with typically developmental controls at $p_{uncorrected}$ <0.0005 and minimum cluster size = 10 voxels (replication analysis).

**Region**	**MNI coordinate**			**SDM**	***P < 0.005***	**Voxels**	**Cluster breakdown**
	x	y	z	***Z* score**	***(Uncorrected)***		**(Voxels)**
**Dyslexia > TDCs**							
No cluster found							
**Dyslexia < TDCs**							
No cluster found							

Abbreviations: TDCs, typically developing controls; SDM, Seed-based d Mapping.

BRAIN VOLUME SUB-ANALYSIS

Table S7. Brain clusters of gray matter variation in dyslexia compared with typically developmental controls at $p_{uncorrected}$ <0.0005 and minimum cluster size = 10 voxels (brain volume sub-analysis).

**Region**	**MNI coordinate**			**SDM**	***P < 0.005***	**Voxels**	**Cluster breakdown**
	x	y	z	***Z* score**	***(Uncorrected)***		**(Voxels)**
**Dyslexia > TDCs**							
No cluster found							
**Dyslexia < TDCs**							
Left superior temporal gyrus (BA 38)	-46	10	-14	-3.578	0.0001	207	Left STG (181)<br>Left MTG (26)
Right lobule VI (Cerebellum)	36	-58	-24	-2.839	0.002	34	Right lobule VI (22)<br>Right crus I (12)
Middle cerebellar peduncles	-24	-44	-32	-2.715	0.003	14	Middle cerebellar peduncles (14)

Abbreviations: TDCs, typically developing controls; BA, Brodmann area; MNI, Montreal Neurological Institute; SDM, Seed-based d Mapping; STG, superior temporal gyrus; MTG, middle temporal gyrus.

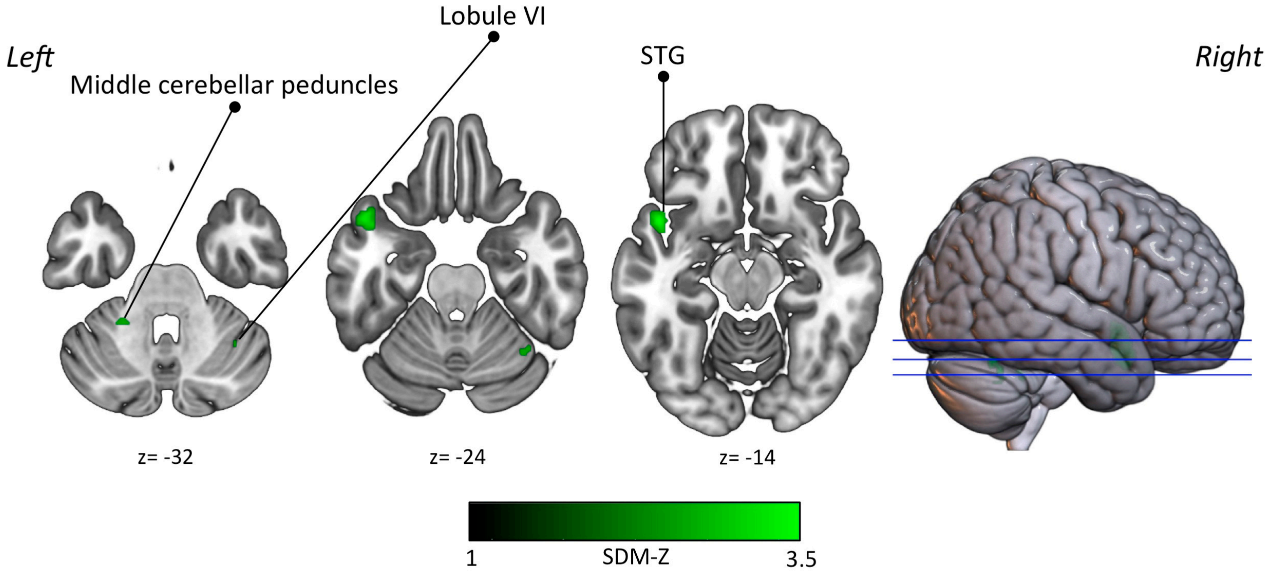

Figure S3. Brain clusters of gray matter variation in dyslexia compared with typically developmental controls at $p_{uncorrected}$ <0.0005 and minimum cluster size = 10 voxels (brain volume sub-analysis).

The PSI-SDM map is visualized as six axial slices (2-D cortical, subcortical, and cerebellar view). Colors from dark to light green represent voxels with a common pattern of neuroanatomical reduction (gray matter in dyslexia < typically developmental controls). Brain slices are in neurological convention (i.e. Right is right, Left is left). STG, superior temporal gyrus.

Table S8. Brain clusters of gray matter variation in attention-deficit/hyper-activity disorder compared with typically developmental controls at $p_{uncorrected}$ <0.0005 and minimum cluster size = 10 voxels (brain volume sub-analysis).

Region	MNI coordinate			SDM	*P < 0.005*	Voxels	Cluster breakdown
	x	y	z	*Z* score	*(Uncorrected)*		(Voxels)
**ADHD > TDCs**							
No cluster found							
**ADHD < TDCs**							
Left crus II (Cerebellum)	-22	-78	-36	-3.569	0.0001	693	Left crus II (372)<br>Left crus I (199)<br>Left lobule VIIB (52)<br>Left lobule VI (36)<br>Left lobule VIII (34)
Left parahippocampal gyrus (BA 36)	-20	-12	-24	-4.468	0.000003	372	Left parahippocampus (247)<br>Left hippocampus (55)<br>Left medial cingulum (33)<br>Left fusiform gyrus (22)<br>Left amygdala (15)
Right caudate nucleus	16	14	10	-4.509	0.000003	276	Right caudate (276)
Left cuneus cortex (BA 17)	-10	-98	14	-4.362	0.000006	227	Left cuneus (135)<br>Left SOG (78)<br>Left MOG (7)
Left caudate nucleus	-12	20	6	-4.213	0.00001	157	Left caudate (152)<br>Left striatum (5)
Right gyrus rectus (BA 11)	10	38	-24	-3.414	0.0003	165	Right gyrus rectus (153)<br>Right SFG (12)
Right lenticular nucleus (Putamen)	30	-2	-4	-3.400	0.0003	136	Right putamen (108)<br>Right striatum (28)
Left anterior cingulate cortex (BA 32)	-4	40	4	-3.218	0.0006	124	Left ACC (109)<br>Right ACC (15)
Right lobule IX (Cerebellum)	10	-50	-50	-3.343	0.0004	104	Right lobule IX (104)
Left lobule IX (Cerebellum)	-10	-48	-52	-3.566	0.0001	58	Left lobule IX (58)

Right superior temporal gyrus (BA 38)	28	8	-26	-3.067	0.0007	38	Right STG (26)<br>Right parahippocampal (12)
Right parahippocampal gyrus (BA 20)	30	-26	-24	-3.013	0.001	17	Right parahippocampal (17)
Left supplementary motor area (BA 6)	-8	-12	50	-3.645	0.0001	14	Left SMA (11)

Abbreviations: ADHD, attention-deficit/hyper-activity disorder; TDCs, typically developing controls; BA, Brodmann area; MNI, Montreal Neurological Institute; SDM, Seed-based d Mapping; SOG, superior occipital gyrus; MOG, middle occipital gyrus; ACC, anterior cingulate cortex; STG, superior temporal gyrus; SMA, supplementary motor area.

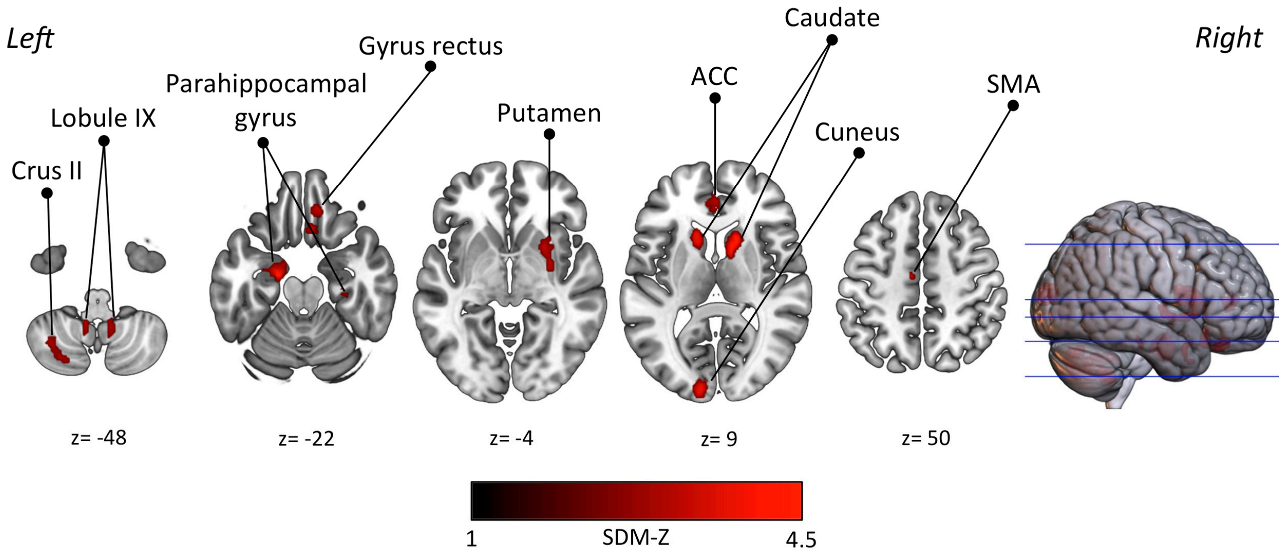

Figure S4. Brain clusters of gray matter variation in attention-deficit/hyper-activity disorder compared with typically developmental controls at $p_{uncorrected}$ <0.0005 and minimum cluster size = 10 voxels (brain volume sub-analysis).

The PSI-SDM map is visualized as six axial slices (2-D cortical, subcortical, and cerebellar view). Colors from dark to light red represent voxels with a common pattern of neuroanatomical reduction (gray matter in attention-deficit/hyper-activity disorder < typically developmental controls). Brain slices are in neurological convention (i.e. Right is right, Left is left). ACC, anterior cingulate cortex; SMA, supplementary motor area.

References

Ahrendts, J., Rüsch, N., Wilke, M., Philipsen, A., Eickhoff, S.B., Glauche, V., Perlov, E., Ebert, D., Hennig, J., Tebartz Van Elst, L., 2011. Visual cortex abnormalities in adults with ADHD: A structural MRI study. World J. Biol. Psychiatry 12, 260–270. https://doi.org/10.3109/15622975.2010.518624

Amico, F., Stauber, J., Koutsouleris, N., Frodl, T., 2011. Anterior cingulate cortex gray matter abnormalities in adults with attention deficit hyperactivity disorder: A voxel-based morphometry study. Psychiatry Res. - Neuroimaging 191, 31–35. https://doi.org/10.1016/j.pscychresns.2010.08.011

Bonath, B., Tegelbeckers, J., Wilke, M., Flechtner, H.H., Krauel, K., 2018. Regional Gray Matter Volume Differences Between Adolescents With ADHD and Typically Developing Controls: Further Evidence for Anterior Cingulate Involvement. J. Atten. Disord. 22, 627–638. https://doi.org/10.1177/1087054715619682

Bralten, J., Greven, C.U., Franke, B., Mennes, M., Zwiers, M.P., Rommelse, N.N.J., Hartman, C., van der Meer, D., O'Dwyer, L., Oosterlaan, J., Hoekstra, P.J., Heslenfeld, D., Arias-Vasquez, A., Buitelaar, J.K., 2016. Voxel-based morphometry analysis reveals frontal brain differences in participants with ADHD and their unaffected siblings. J. Psychiatry Neurosci. 41, 272–279. https://doi.org/10.1503/jpn.140377

Brambati, S.M., Termine, C., Ruffino, M., Stella, G., Fazio, F., Cappa, S.F., Perani, D., 2004. Regional reductions of gray matter volume in familial dyslexia. Neurology 63, 742–745. https://doi.org/10.1212/01.WNL.0000134673.95020.EE

Brieber, S., Neufang, S., Bruning, N., Kamp-Becker, I., Remschmidt, H., Herpertz-Dahlmann, B., Fink, G.R., Konrad, K., 2007. Structural brain abnormalities in adolescents with autism spectrum disorder and patients with attention deficit/hyperactivity disorder. J. Child Psychol. Psychiatry Allied Discip. 48, 1251–1258. https://doi.org/10.1111/j.1469-7610.2007.01799.x

Brown, W.E., Eliez, S., Menon, V., Rumsey, J.M., White, C.D., Reiss, A.L., 2001. Preliminary evidence of widespread morphological variations of the brain in dyslexia. Neurology 56, 781–783. https://doi.org/10.1212/WNL.56.6.781

Carmona, S., Vilarroya, O., Bielsa, A., Trèmols, V., Soliva, J.C., Rovira, M., Tomàs, J., Raheb, C., Gispert, J.D., Batlle, S., Bulbena, A., 2005. Global and regional gray matter reductions in ADHD: A voxel-based morphometric study. Neurosci. Lett. 389, 88–93. https://doi.org/10.1016/j.neulet.2005.07.020

Depue, B.E., Burgess, G.C., Bidwell, L.C., Willcutt, E.G., Banich, M.T., 2010. Behavioral performance predicts grey matter reductions in the right inferior frontal gyrus in young adults with combined type ADHD. Psychiatry Res. - Neuroimaging 182, 231–237. https://doi.org/10.1016/j.pscychresns.2010.01.012

Eckert, M.A., Berninger, V.W., Vaden, K.I., Gebregziabher, M., Tsu, L., 2016. Gray matter features of reading disability: A combined meta-analytic and direct analysis approach. eNeuro 3, 11296–11301. https://doi.org/10.1523/ENEURO.0103-15.2015

Eckert, M.A., Leonard, C.M., Wilke, M., Eckert, M., Richards, T., Richards, A., Berninger, V., 2005. Anatomical signatures of dyslexia in children: Unique information from manual and voxel based morphometry brain measures. Cortex 41, 304–315. https://doi.org/10.1016/S0010-9452(08)70268-5

Evans, T.M., Flowers, D.L., Napoliello, E.M., Eden, G.F., 2014. Sex-specific gray matter volume differences in females with developmental dyslexia. Brain Struct. Funct. https://doi.org/10.1007/s00429-013-0552-4

He, N., Li, F., Li, Y., Guo, L., Chen, L., Huang, X., Lui, S., Gong, Q., 2015. Neuroanatomical deficits correlate with executive dysfunction in boys with attention deficit hyperactivity disorder. Neurosci. Lett. 600, 45–49. https://doi.org/10.1016/j.neulet.2015.05.062

Hoeft, F., Meyler, A., Hernandez, A., Juel, C., Taylor-Hill, H., Martindale, J.L., McMillon, G., Kolchugina, G., Black, J.M., Faizi, A., Deutsch, G.K., Wai, T.S., Reiss, A.L., Whitfield-Gabrieli, S., Gabrieli, J.D.E., 2007. Functional and morphometric brain dissociation between dyslexia and reading ability. Proc. Natl. Acad. Sci. U. S. A. 104, 4234–4239. https://doi.org/10.1073/pnas.0609399104

Iannaccone, R., Hauser, T.U., Ball, J., Brandeis, D., Walitza, S., Brem, S., 2015. Classifying adolescent attention-deficit/hyperactivity disorder (ADHD) based on functional and structural imaging. Eur. Child Adolesc. Psychiatry 24, 1279–1289. https://doi.org/10.1007/s00787-015-0678-4

Jednoróg, K., Marchewka, A., Altarelli, I., Monzalvo Lopez, A.K., van Ermingen-Marbach, M., Grande, M., Grabowska, A., Heim, S., Ramus, F., 2015. How reliable are gray matter disruptions in specific reading disability across multiple countries and languages? Insights from a large-scale voxel-based morphometry study. Hum. Brain Mapp. 36, 1741–1754. https://doi.org/10.1002/hbm.22734

Johnston, B.A., Mwangi, B., Matthews, K., Coghill, D., Konrad, K., Steele, J.D., 2014. Brainstem abnormalities in attention deficit hyperactivity disorder support high accuracy individual diagnostic classification. Hum. Brain Mapp. 35, 5179–5189. https://doi.org/10.1002/hbm.22542

Kappel, V., Lorenz, R.C., Streifling, M., Renneberg, B., Lehmkuhl, U., Ströhle, A., Salbach-Andrae, H., Beck, A., 2015. Effect of brain structure and function on reward anticipation in children and adults with attention deficit hyperactivity disorder combined subtype. Soc. Cogn. Affect. Neurosci. 10, 945–951. https://doi.org/10.1093/scan/nsu135

Kobel, M., Bechtel, N., Specht, K., Klarhöfer, M., Weber, P., Scheffler, K., Opwis, K., Penner, I.K., 2010. Structural and functional imaging approaches in attention deficit/hyperactivity disorder: Does the temporal lobe play a key role? Psychiatry Res. - Neuroimaging 183, 230–236. https://doi.org/10.1016/j.pscychresns.2010.03.010

Kronbichler, M., Wimmer, H., Staffen, W., Hutzier, F., Mair, A., Ladurner, G., 2008. Developmental dyslexia: Gray matter abnormalities in the occipitotemporal cortex. Hum. Brain Mapp. 29, 613–625. https://doi.org/10.1002/hbm.20425

Kumar, U., Arya, A., Agarwal, V., 2017. Neural alterations in ADHD children as indicated by voxel-based cortical thickness and morphometry analysis. Brain Dev. 39, 403–410. https://doi.org/10.1016/j.braindev.2016.12.002

Lim, L., Marquand, A., Cubillo, A.A., Smith, A.B., Chantiluke, K., Simmons, A., Mehta, M., Rubia, K., 2013. Disorder-Specific Predictive Classification of Adolescents with Attention Deficit Hyperactivity Disorder (ADHD) Relative to Autism Using Structural Magnetic Resonance Imaging. PLoS One 8, 1–10. https://doi.org/10.1371/journal.pone.0063660

Liu, L., You, W., Wang, W., Guo, X., Peng, D., Booth, J., 2013. Altered brain structure in Chinese dyslexic children. Neuropsychologia 51, 1169–1176. https://doi.org/10.1016/j.neuropsychologia.2013.03.010

Maier, S., Perlov, E., Graf, E., Dieter, E., Sobanski, E., Rump, M., Warnke, A., Ebert, D., Berger, M., Matthies, S., Philipsen, A., Tebartz van Elst, L., 2016. Discrete Global but No Focal Gray Matter Volume Reductions in Unmedicated Adult Patients With Attention-Deficit/Hyperactivity Disorder. Biol. Psychiatry 80, 905–915. https://doi.org/10.1016/j.biopsych.2015.05.012

McAlonan, G.M., Cheung, V., Cheung, C., Chua, S.E., Murphy, D.G.M., Suckling, J., Tai, K.S., Yip, L.K.C., Leung, P., Ho, T.P., 2007. Mapping brain structure in attention deficit-hyperactivity disorder: A voxel-based MRI study of regional grey and white matter volume. Psychiatry Res. - Neuroimaging 154, 171–180. https://doi.org/10.1016/j.pscychresns.2006.09.006

Montes, L.G.A., Ricardo-Garcell, J., de la Torre, L.B., Alcántara, H.P., García, R.B.M., Fernández-Bouzas, A., Acosta, D.Á., 2010. Clinical correlations of grey matter reductions in the caudate nucleus of adults with attention deficit hyperactivity disorder. J. Psychiatry Neurosci. 35, 238–246. https://doi.org/10.1503/jpn.090099

Moreno-Alcázar, A., Ramos-Quiroga, J.A., Radua, J., Salavert, J., Palomar, G., Bosch, R., Salvador, R., Blanch, J., Casas, M., McKenna, P.J., Pomarol-Clotet, E., 2016. Brain abnormalities in adults with Attention Deficit Hyperactivity Disorder revealed by voxel-based morphometry. Psychiatry Res. - Neuroimaging 254, 41–47. https://doi.org/10.1016/j.pscychresns.2016.06.002

Onnink, A.M.H., Zwiers, M.P., Hoogman, M., Mostert, J.C., Kan, C.C., Buitelaar, J., Franke, B., 2014. Brain alterations in adult ADHD: Effects of gender, treatment and comorbid depression. Eur. Neuropsychopharmacol. 24, 397–409. https://doi.org/10.1016/j.euroneuro.2013.11.011

Overmeyer, S., Bullmore, E.T., Suckling, J., Simmons, A., Williams, S.C.R., Santosh, P.J., Taylor, E., 2001. Distributed grey and white matter deficits in hyperkinetic disorder: MRI evidence for anatomical abnormality in an attentional network. Psychol. Med. 31, 1425–1435. https://doi.org/10.1017/s0033291701004706

Pernet, C., Andersson, J., Paulesu, E., Demonet, J.F., 2009. When all hypotheses are right: A multifocal account of dyslexia. Hum. Brain Mapp. 30, 2278–2292. https://doi.org/10.1002/hbm.20670

Roman-Urrestarazu, A., Lindholm, P., Moilanen, I., Kiviniemi, V., Miettunen, J., Jääskeläinen, E., Mäki, P., Hurtig, T., Ebeling, H., Barnett, J.H., Nikkinen, J., Suckling, J., Jones, P.B., Veijola,

J., Murray, G.K., 2016. Brain structural deficits and working memory fMRI dysfunction in young adults who were diagnosed with ADHD in adolescence. Eur. Child Adolesc. Psychiatry 25, 529–538. https://doi.org/10.1007/s00787-015-0755-8

Saad, J.F., Griffiths, K.R., Kohn, M.R., Clarke, S., Williams, L.M., Korgaonkar, M.S., 2017. Regional brain network organization distinguishes the combined and inattentive subtypes of Attention Deficit Hyperactivity Disorder. NeuroImage Clin. 15, 383–390. https://doi.org/10.1016/j.nicl.2017.05.016

Sasayama, D., Hayashida, A., Yamasue, H., Harada, Y., Kaneko, T., Kasai, K., Washizuka, S., Amano, N., 2010. Neuroanatomical correlates of attention-deficit-hyperactivity disorder accounting for comorbid oppositional defiant disorder and conduct disorder. Psychiatry Clin. Neurosci. 64, 394–402. https://doi.org/10.1111/j.1440-1819.2010.02102.x

Seidman, L.J., Biederman, J., Liang, L., Valera, E.M., Monuteaux, M.C., Brown, A., Kaiser, J., Spencer, T., Faraone, S. V., Makris, N., 2011. Gray matter alterations in adults with attention-deficit/hyperactivity disorder identified by voxel based morphometry. Biol. Psychiatry 69, 857–866. https://doi.org/10.1016/j.biopsych.2010.09.053

Silani, G., Frith, U., Demonet, J.F., Fazio, F., Perani, D., Price, C., Frith, C.D., Paulesu, E., 2005. Brain abnormalities underlying altered activation in dyslexia: A voxel based morphometry study. Brain 128, 2453–2461. https://doi.org/10.1093/brain/awh579

Steinbrink, C., Vogt, K., Kastrup, A., Müller, H.P., Juengling, F.D., Kassubek, J., Riecker, A., 2008. The contribution of white and gray matter differences to developmental dyslexia: Insights from DTI and VBM at 3.0 T. Neuropsychologia 46, 3170–3178. https://doi.org/10.1016/j.neuropsychologia.2008.07.015

Sutcubasi Kaya, B., Metin, B., Tas, Z.C., Buyukaslan, A., Soysal, A., Hatiloglu, D., Tarhan, N., 2018. Gray Matter Increase in Motor Cortex in Pediatric ADHD: A Voxel-Based Morphometry Study. J. Atten. Disord. 22, 611–618. https://doi.org/10.1177/1087054716659139

Tamboer, P., Scholte, H.S., Vorst, H.C.M., 2015. Dyslexia and voxel-based morphometry: correlations between five behavioural measures of dyslexia and gray and white matter volumes. Ann. Dyslexia 65, 121–141. https://doi.org/10.1007/s11881-015-0102-2

van Wingen, G.A., van den Brink, W., Veltman, D.J., Schmaal, L., Dom, G., Booij, J., Crunelle, C.L., 2013. Reduced striatal brain volumes in non-medicated adult ADHD patients with comorbid cocaine dependence. Drug Alcohol Depend. 131, 198–203. https://doi.org/10.1016/j.drugalcdep.2013.05.007

Villemonteix, T., De Brito, S.A., Kavec, M., Balériaux, D., Metens, T., Slama, H., Baijot, S., Mary, A., Peigneux, P., Massat, I., 2015. Grey matter volumes in treatment naïve vs. chronically treated children with attention deficit/hyperactivity disorder: A combined approach. Eur. Neuropsychopharmacol. 25, 1118–1127. https://doi.org/10.1016/j.euroneuro.2015.04.015

Villemonteix, T., De Brito, S.A., Slama, H., Kavec, M., Balériaux, D., Metens, T., Baijot, S., Mary, A., Peigneux, P., Massat, I., 2015. Grey matter volume differences associated with gender in children with attention-deficit/hyperactivity disorder: A voxel-based morphometry study. Dev. Cogn. Neurosci. 14, 32–37. https://doi.org/10.1016/j.dcn.2015.06.001

Vinckenbosch, E., Robichon, F., Eliez, S., 2005. Gray matter alteration in dyslexia: Converging evidence from volumetric and voxel-by-voxel MRI analyses. Neuropsychologia 43, 324–331. https://doi.org/10.1016/j.neuropsychologia.2004.06.02

Wai, T.S., Niu, Z., Jin, Z., Perfetti, C.A., Li, H.T., 2008. A structural-functional basis for dyslexia in the cortex of Chinese readers. Proc. Natl. Acad. Sci. U. S. A. 105, 5561–5566. https://doi.org/10.1073/pnas.0801750105

Xia, Z., Hoeft, F., Zhang, L., Shu, H., 2016. Neuroanatomical anomalies of dyslexia: Disambiguating the effects of disorder, performance, and maturation. Neuropsychologia 81, 68–78. https://doi.org/10.1016/j.neuropsychologia.2015.12.003

Yang, P., Wang, P.N., Chuang, K.H., Jong, Y.J., Chao, T.C., Wu, M.T., 2008. Absence of gender effect on children with attention-deficit/hyperactivity disorder as assessed by optimized voxel-based morphometry. Psychiatry Res. - Neuroimaging 164, 245–253. https://doi.org/10.1016/j.pscychresns.2007.12.013

Yang, Y.H., Yang, Y., Chen, B.G., Zhang, Y.W., Bi, H.Y., 2016. Anomalous cerebellar anatomy in Chinese children with dyslexia. Front. Psychol. 7, 1–9. https://doi.org/10.3389/fpsyg.2016.00324
